# SNF2L suppresses nascent DNA gap formation to promote DNA synthesis

**DOI:** 10.1093/nar/gkae903

**Published:** 2024-10-16

**Authors:** Anthony Nelligan, Huzefa Dungrawala

**Affiliations:** Department of Molecular Biosciences, University of South Florida, Tampa, FL 33620, USA; Department of Molecular Biosciences, University of South Florida, Tampa, FL 33620, USA

## Abstract

Nucleosome remodelers at replication forks function in the assembly and maturation of chromatin post DNA synthesis. The ISWI chromatin remodeler SNF2L (or SMARCA1) travels with replication forks but its contribution to DNA replication remains largely unknown. We find that fork elongation is curtailed when SNF2L is absent. SNF2L deficiency elevates replication stress and causes fork collapse due to remodeling activities by fork reversal enzymes. Mechanistically, SNF2L regulates nucleosome assembly to suppress post-replicative ssDNA gap accumulation. Gap induction is not dependent on fork remodeling and PRIMPOL. Instead, gap synthesis is driven by MRE11 and EXO1 indicating susceptibility of nascent DNA to nucleolytic cleavage and resection when SNF2L is removed. Additionally, nucleosome remodeling by SNF2L protects nascent chromatin from MNase digestion and gap induction highlighting a critical role of SNF2L in chromatin assembly post DNA synthesis to maintain unperturbed replication.

## Graphical Abstract

**Graphical Abstract:**
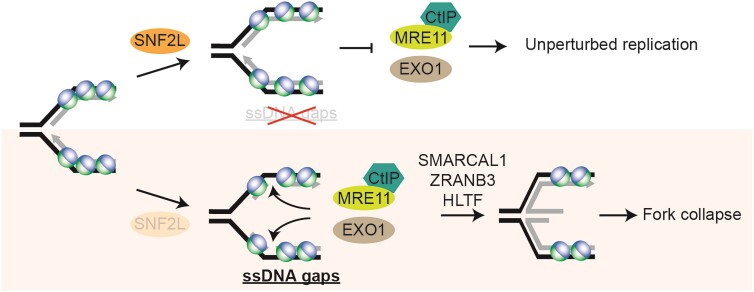

## Introduction

Successful passage of a replication fork entails displacement of parental nucleosomes in front of the fork and rapid reassembly of nucleosomes behind the fork (1,2). Nucleosome core particles consist of roughly 147 bp of double stranded DNA wrapped around a histone octamer that comprises a central H3–H4 tetramer flanked by two H2A–H2B dimers. Reassembly of nucleosomes on the newly synthesized daughter molecules occurs by a combination of de novo replication-dependent nucleosome assembly to provide new unmodified histones and recycling of old histones by parental histone segregation (3,4). To help successfully re-establish chromatin post DNA synthesis and prevent loss in heritability due to replication-coupled dilution, several factors associate with the replisome including histone chaperones, histone modifying enzymes and nucleosome remodelers.

ATP-dependent chromatin remodelers are categorized into four subfamilies based on their domain architecture and biological function- switch/sucrose non-fermentable (SWI/SNF), imitation switch (ISWI), inositol-requiring mutant 80 (INO80) and chromodomain helicase DNA-binding (CHD) (5,6). All remodelers share a conserved ATPase domain which impart the ability to translocate DNA along histone surfaces. In comparison, distinct nucleosomal epitopes, recognition domains and association with accessory subunits regulate binding of remodelers to distinct nucleosomal targets to elicit divergent biological outcomes. The ISWI and CHD subfamily members are generally involved in repositioning of assembled nucleosomes to generate regularly-spaced nucleosomal arrays (7–9). By sliding or evicting nucleosomes, the SWI/SNF subfamily helps regulate DNA accessibility to DNA repair and transcription machineries (10). The INO80 subfamily promotes nucleosomal editing by removal and incorporation of canonical or variant histones to assist in DSB repair and checkpoint response (11). Thus, by modulating histone-DNA contacts, a diverse set of chromatin remodelers help conduct nucleosomal assembly, editing and accessibility.

Mammalian ISWI subfamily comprises remodelers SNF2L (also called SMARCA1) and SNF2H (also called SMARCA5) that form chromatin remodeling complexes with accessory subunits to regulate diverse functions including development, transcriptional regulation, DNA replication and repair (5,12–14). In addition to the conserved catalytic DEXD ATPase domain and a helicase domain, the ISWI remodelers harbor a distinct HAND-SANT-SLIDE (HSS) domain at the C-terminus responsible for unmodified histone H4 tail recognition and DNA binding (15). Although the ATPase subunits can reposition nucleosomes independently of a complex, the presence of regulatory subunits help differentially regulate periodicity in nucleosomal spacing (7,16). SNF2L associates with BPTF, RBBP4 and RBBP7 to form the nucleosome remodeling factor (NURF) complex (17,18) and with CECR2 to form CECR2-containing remodeling factor (CERF) complex (19) respectively. NURF complex has been demonstrated to function in transcriptional activation in flies (20,21) and neurite outgrowth in humans (22,23). Recent study shows all ISWI regulatory subunits exist as stable complexes with either SMARCA1 and SMARCA5 and demonstrate that either remodeler exhibits nucleosome sliding activity independently or in combination with the regulatory complex subunits (24). Fluorescence kinetic analyses in human cells also reveal that residence times of SNF2L and paralog SNF2H on chromatin increase by 40–70% in S-phase compared to G1/2 phases indicating active contribution of these remodelers to DNA replication (25). Indeed, yeast Isw2 and Ino80 play critical roles in promoting replication fork progression by bindings to sites of replication (26). Human ISWI ortholog SNF2H in a complex with ACF1 and WSTF plays essential roles in replication through heterochromatin (27,28). SNF2L and the related NURF complex subunits also travel with replication forks (29,30). SNF2L absence causes replication associated increase in DNA damage signaling implicating a critical function of SNF2L at active replication forks (31).

The fidelity of DNA replication is under constant threat from various endogenous and exogenous sources that block ongoing replication forks. Multiple DNA damage tolerance (DDT) pathways help overcome replication obstacles and aid in restart including replication fork reversal and fork repriming (32). Fork reversal converts replication forks into four-way junctions by annealing the nascent strands and occurs in response to a wide variety of DNA replication obstacles and structural abnormalities (33). DNA translocases SMARCAL1, HLTF and ZRANB3 catalyze replication fork reversal in cooperation with RAD51 to slow ongoing forks (34,35). While fork reversal can promote error-free repair of DNA lesions, fork regression must be tightly regulated since reversed forks are prone to nucleolytic degradation or cleavage causing fork collapse into double strand breaks (DSBs) (36–40). Fork repriming reinitiates replication downstream of the lesion thereby promoting fork restart. In mammals, repriming is performed by Primase and DNA-directed Polymerase (PRIMPOL) that catalyzes both DNA priming and polymerization (41,42). Unlike translesion DNA synthesis (TLS) that uses the damaged template for DNA synthesis (43), PRIMPOL synthesizes DNA beyond the lesion resulting in generation of single-stranded DNA (ssDNA) gaps that are either filled by alternate DDT mechanisms, such as template-switching and TLS (32), or converted to breaks (44). Replication gaps can also originate from defective Okazaki fragment maturation (45) or expanded by resection nucleases MRE11 and EXO1 (46,47). The choice of DDT mechanism is dictated by several factors such as type of DNA lesion and genetic context and is tightly regulated to maintain genome integrity. For instance, cells exhibit preference towards either PRIMPOL-mediated repriming or replication fork reversal in response to fork stalling conditions (48–50).

Here, we show that SNF2L promotes fork progression by suppressing accumulation of replication gaps. Loss of SNF2L slows replication speed and induces break formation that is dependent on MUS81. Depleting fork reversal enzymes abrogates occurrence of DNA breaks and rescues elongation defects in SNF2L-deficient cells indicating that fork remodeling events curtail replication when SNF2L is depleted. Characterization of replication stress markers reveals existence of ssDNA gaps in nascent DNA when SNF2L is removed. The accumulation of replication gaps is not dependent on fork reversal enzymes and PRIMPOL but does become PRIMPOL-dependent when fork reversal is abolished. Notably, depleting MRE11 and EXO1 restores fork elongation and suppresses gap formation indicating that nascent DNA is prone to resection in SNF2L-deficient conditions. Furthermore, blocking endonucleolytic activity of MRE11 or depleting CtIP restricts gap accumulation and rescues fork elongation rates indicating that SNF2L absence triggers gap induction by nucleolytic activity of MRE11. Mechanistically, SNF2L promotes chromatin assembly on nascent DNA to prevent aberrant induction and processing of gaps by nucleases. Our findings highlight SNF2L as a mediator of replication fork progression that blocks gap synthesis during genome duplication.

## Materials and methods

### Cell lines

U2OS, HeLa and HEK293T cell lines were cultured in DMEM with 7.5% FBS. hTERT-RPE-1 cell lines were cultured in DMEM: F12 with 7.5% FBS. All cell lines were incubated at 37°C with 5% CO_2_. SNF2L knockout (SNF2L KO) U2OS cell lines used in this study were generated using CRISPR/Cas9 technology. U2OS cells were co-transfected with pair of pSpCas9-(BB)-2A-Puro vectors containing guide RNAs targeting Exon 1 (GGATGCGACCGCCACTATCGG and CGGAGAAGGGCGAGAAGAAG) and selected with puromycin. After 48 h in selection media, surviving cells were serially diluted to clonal populations. Single cell colonies were isolated and SNF2L expression in multiple clones was evaluated by immunoblotting. SNF2L KO cells stably expressing SNF2L cDNA constructs were generated by transfecting wild-type or K214A cDNA plasmids with FUGENE HD. 48 h after transfection, cells were selected with 400μg/μl G418 sulfate (Gibco, 10131035) until no viable cells were detectable on control plate. Following selection, surviving cells were maintained in DMEM + 7.5% FBS with 200 μg/μl G418 sulfate. FBH1 knockout (KO) cell lines were generated as previously reported (51).

### Transfections

Plasmid DNA transfections were performed with FUGENE HD (Promega) in U2OS cells. siRNA transfections were performed with Dharmafect1 (Dharmacon) in U2OS cells and RNAimax (Thermo Fisher Scientific) in hTERT-RPE-1, HEK293T and HeLa cells. Unless otherwise indicated, all assays were performed 72 h post transfection.

### Immunofluorescence analysis

Cells were treated with thymidine analogs and/or drugs where indicated, rinsed with DPBS, and pre-extracted using 0.5% Triton X-100 before fixation with either 3% paraformaldehyde/2% sucrose or 4% paraformaldehyde. After blocking with 5% BSA/PBS, cells were incubated with primary and secondary antibodies. Coverslips were mounted using Prolong Gold with DAPI (Invitrogen). For nascent ssDNA quantification in native conditions, cells were pre-treated where indicated and labeled with 10μM IdU for 20 min, pre-extracted using 0.5% Triton X-100 followed by fixation and immunostaining. For quantifying parental ssDNA in native conditions, cells were pre-treated where indicated and labeled with 10 μM IdU for 22 h, after which IdU containing media was washed out and cells were chased in IdU-free media for 2 h followed by pre-extraction using 0.5% Triton X-100, fixation and immunostaining. IdU was detected using Mouse anti-BrdU (1:50; BD Biosciences) and Goat anti-mouse Alexa 488 (1:500; Invitrogen). For all HU treatments, cells were pulsed with 10μM EdU for 10 min followed by 4mM HU treatment for 4 h. For phospho-histone H3 (Ser 10) counterstaining experiments with EdU, cells were either untreated or treated with 0.4 μM aphidicolin for 17 h followed by release into fresh media with 10μM EdU for 30 min. For quantification of nuclear PAR (poly ADP ribose) intensities, cells were treated with 10μM PARGi (Selleckchem, PDD00017273) for 30 min and pulsed with 10μM EdU in the final 10 min of PARGi treatment. To detect replicating cells, EdU was detected by click chemistry using Alexa Fluor 488 or 594 Azide (Invitrogen). Following antibodies were used for immunostaining to quantify nuclei intensity: Histone H2A.X Ser139 (1:9000, Millipore Sigma, 05-636-25), RPA32 (1:200, Abcam, ab2175), mono/poly-ADP-ribose Mab (1:200, Cell Signaling, 83732), phospho histone H3 Ser 10 (1:200, Cell Signaling Technology 9701S), anti-Rabbit Alexa Fluor 594 (1:2000, Thermo Fisher Scientific, A11037) and anti-Mouse Alexa Fluor 594 (1:2000, Thermo Fisher Scientific, A11005). For proximity ligation analyses (PLA), *in situ* Protein Interaction with Nascent DNA Replication Forks (SIRF) assay (52) was performed using Duolink In Situ Mouse/Rabbit Kit (Sigma-Aldrich). Cells were labeled with 125 μM EdU for 10 min prior to fixation, click reaction and immunostaining. Following antibodies were used to detect PLA foci: SMARCA1 (1:150, Cell Signaling Tech, 9450) and anti-biotin (1:200, Jackson ImmunoResearch, 200–002-211). Image acquisition was performed on a Keyence BZ-X810 fluorescence microscope using a 20× objective (0.75NA). Cell profiler or BZ-X810 analyzer was used to measure mean nuclei intensity. ImageJ was used to quantify PLA foci. All measurements of mean nuclei intensities are depicted in arbitrary units.

### DNA fiber analysis by spreading

DNA fiber labeling analysis was performed as described previously (53). To measure fork speeds in unchallenged conditions, cells were pulsed sequentially with 20 μM CldU and 100 μM IdU for 30 min each. Cells were harvested, lysed on slides and DNA was stretched by tilting the slides. DNA was then fixed using a 3:1 solution of methanol: acetic acid and stored at −20°C overnight. Next day, DNA was rehydrated in PBS prior to denaturation with 2.5N HCl for 40 min, blocked in PBS containing 0.1% Triton X-100 and 10% goat serum for 1 h, stained with primary antibodies for 2 h to recognize IdU (1:10, anti-BrdU, BD Biosciences, BD347580) and CldU (1:100, anti-BrdU, Abcam, ab6326) followed by staining with secondary antibodies (1:350, Goat anti-rat Alexa Fluor 594, Thermo Fisher Scientific, A11007) and (1:175, Goat anti-mouse Alexa Fluor 488, Thermo Fisher Scientific, A11029) for 1 h. Images were acquired using a 60× oil objective (1.4 NA) on Keyence BZ-X810 and fiber lengths were analyzed using ImageJ.

### ssDNA gap analysis by S1 nuclease fiber assay

DNA fiber labeling was performed as described above with the following modifications to detect ssDNA gaps (54). Cells were sequentially pulsed with 20 μM CldU for 20 min followed by 100 μM IdU for 60 min. Where indicated, cells were pretreated for 2 h with 50μM mirin (Selleck Chemicals, S8096), 100 μM PFM-01 (Selleck Chemicals, S3549), 50 μM C5 (MedChem Express 35973-25-2) or 10μM PARP inhibitor Olaparib (Selleck Chemicals, S8096). For chase experiments, cells were pulsed with 10 μM Thymidine following IdU washout and incubated for the indicated times. Post-labeling, cells were treated with cytoskeleton CSK100 buffer (100 mM NaCl, 10 mM MOPS pH 7, 3 mM MgCl_2_, 300 mM sucrose and 0.5% Triton X-100 in water) at room temperature for 8 min. Cells were rinsed sequentially with HBSS and S1 buffers, then treated with S1 nuclease for 30 min at 37°C. Untreated nuclei were left in S1 buffer for the duration. Nuclei were harvested by scraping, lysed, and DNA was stretched on slides as described in DNA fiber analysis. Images were acquired using a 60× oil objective (1.4 NA) on Keyence BZ-X810 and fiber lengths were analyzed using ImageJ.

### DNA combing assay

DNA combing was performed following Genomic Vision manufacturer's instructions. Briefly, cells were sequentially labeled with 20 μM CldU and 100 μM IdU for 30 min, harvested, and embedded in low-melt agarose plugs. Plugs were treated overnight with Proteinase K, washed thoroughly, and treated overnight with beta-agarase. After agarase treatment, free DNA was transferred to reservoir containing MES buffer at pH 5.5 and allowed to acclimate to room temperature before combing with GenomicVision coverslips and FiberComb apparatus. Combed coverslips were held at 60°C for 2 h before proceeding with immunostaining with primary antibodies to detect CldU (1:31.25, Abcam, ab6326), IdU (1:6.25, BD Biosciences, BD347580), and DNA (1:12.5, Autoanti-ssDNA, DSHB) followed by secondary antibodies anti-Rat Cy5 (1:12.5, Abcam, ab6565), anti-Mouse (1:12.5, Abcam, ab97035) and anti-mouse BV 480 (1:12.5, Jackson Immunoresearch, 115-685-166). Slides were scanned using FiberVision S automated scanner (Genomic Vision). 40× objective magnification was used with 8-bit depth for each channel. Data were extracted and analysis of replication dynamics was performed using FiberStudio 2.0 software (Genomic Vision). For S1 nuclease-DNA combing assays, 1 mM zinc acetate and either 40 U/ml S1 nuclease or S1 buffer (Thermo Scientific; EN0321) was added to MES buffer following the beta-agarase treatment (55).

### Neutral comet assay

DNA breaks were detected as per manufacturer's instructions using Biotechne comet assay kit with slight modifications. Briefly, cells were harvested, rinsed with PBS, resuspended in low melting agarose and spread evenly on the slide. After brief storage at 4°C, slides were incubated in lysis buffer (0.5% SDS, 200 mM Tris–Cl (pH 7.4), 50 mM EDTA) for 1 h at RT. Slides were immersed in cold TAE buffer for 30 min and subjected to electrophoresis for 40 min. Following incubation in DNA precipitation solution, slides were dried and stained with SYBR Green solution. Tail moments were scored using Comet Score software (Tritek) and data presented as box and whisker plots using 10–90 percentile.

### Flow cytometry

For cell cycle analysis, cells were harvested and processed in accordance with manufacturer's instructions (Abcam; ab287852). Briefly, cells were spun down and washed, fixed with 70% ethanol, washed, and resuspended in staining solution. Stained cells were filtered into a flow cytometry tube for flow cytometry analyses. Analyses were carried out on BD FACSCanto II.

### Whole cell lysate extraction and immunoblotting

To obtain whole-cell lysates, cell pellets were incubated on ice for 30 min in lysis buffer (50 mM Tris–Cl pH = 7.4, 1% NP-40, 150 mM NaCl, 0.1% SDS, 0.5% sodium deoxycholate, 1 mM sodium orthovanadate, 1 mM aprotinin, 1 mM leupeptin, 1 mM DTT, 1 mM PMSF) supplemented with Pierce universal nuclease and 1 mM MgCl_2_. After centrifugation for 30 min, supernatant was isolated and quantified prior to immunoblotting with the following antibodies: SMARCA1 (1:1000, Cell Signaling Tech, 9450), SMARCA5 (1:500, Novus Bio, NB100-55301), RECQ1 (1:500, Abcam, ab172607), MUS81 (1:500, Santa Cruz, sc-53382), CtIP (1:500, Santa Cruz, sc-271339), BPTF (1:500, Millipore Sigma, ABE24), PCNA (1:200, Santa Cruz, sc-56), MRE11 (1:500, Cell Signaling Tech, 4895S), DNA2 (1:500, Thermo Fisher Scientific, PA5-77943), SMARCAL1 (1:500, Santa Cruz, sc-376377), ZRANB3 (1:500, Bethyl, A303-033A), HLTF (1:1000, Abcam, ab183042), CCDC111/PRIMPOL (1:1000, Proteintech, 28924–1-AP), EXO1 (1:500, Bethyl, A302-640A), Phospho CHK1 (S317) (1:200, Bethyl, A304-673A), CHK1 (1:500, Santa Cruz, sc-8408), RPA32 (S33) (1:200, Bethyl, A300-246A), RPA32 (1:1000, Abcam, ab2175), KU70 (1:1000, Abcam, ab92450), 800CW anti-Rabbit (1:20 000, LI-COR, 926-32213) and 800CW anti-Mouse (1:20 000, LI-COR, 926-32212).

### MNase assays

To examine genomic MNase sensitivity, cells were pelleted and crosslinked with 1% formaldehyde in PBS for 10 min at room temperature (RT). Crosslinking was quenched by the addition of 2.5 M glycine for 5 min on tube rotator at RT. After quenching, cells were rinsed with PBS, snap-frozen in liquid nitrogen, and stored overnight at −80°C. The following day, cell pellets were thawed and re-suspended in cell lysis buffer (10 mM Tris pH 7.4, 10 mM NaCl, 2 mM MgCl_2_, 0.5% NP-40, 1 mM aprotinin, 1 mM leupeptin, 1 mM DTT, 1 mM PMSF and 3 μM CaCl_2_) and lysed for 15 min at 4°C with gentle rotation. MNase was aliquoted into pre-chilled microcentrifuge tubes and combined with cell lysate for 8 min at 37°C and quenched with 10 mM EDTA promptly following digestion. Digested lysates were treated with RNase A for 2 h at 37°C, moved to 55°C with the addition of 0.01% SDS and Proteinase K overnight. The following day, DNA was purified using a phenol–chloroform extraction. DNA was quantified and equal masses of DNA were run on a 1.5% agarose gel in TBE to examine nucleosomal banding patterns. To examine nascent DNA sensitivity to MNase by IF, cells were seeded on coverslips the day before the experiment. Cells were pulse labeled with 10 μM EdU for 30 min, chased into thymidine where indicated, and permeabilized using 0.5% Triton-X100 buffer for 10 minutes at 4°C. Cells were rinsed gently with PBS and treated with 100 U MNase in MNase buffer (0.32 M sucrose, 50 mM Tris–Cl pH 7.5, 4 mM MgCl_2_, 100 uM PMSF) for 30 min at RT. Cells were rinsed gently with PBS and then incubated with 0.5% Triton-X100 supplemented with 5 mM EGTA to quench MNase activity by chelation. Cells were then fixed with 4% PFA, rinsed with PBS, and blocked with 5% BSA/PBS for 15 min at RT. EdU was detected via Click conjugation of Alexa Fluor 488.

### Oligos and plasmids

All siRNAs used in this study are listed in Supplementary Table S1. pCMV6-EV (PS100001) was used as a negative control for plasmid transfections. SNF2L cDNA (RC239926) expressing transcript variant 3 which lacks exon 13 was purchased from Origene. SNF2L K214A mutant was created per manufacturer's instructions using the QuikChange II XL Site-Directed Mutagenesis kit (Agilent, 200521). Following primers were used to introduce the mutations: Forward primer- 5′-CAAAGCAATTGTTTGTAAAGTTGCCCCAAGGCCCATTTCATCAGC-3′ and reverse primer- 5′-GCTGATGAAATGGGCCTTGGGGCAACTTTACAAACAATTGCTTTG-3′. Mutant constructs were confirmed by whole-plasmid sequencing and expression validated by immunoblotting.

### Proliferation and viability assays

For cell proliferation analysis, U2OS cells were transfected with the indicated siRNAs in triplicate. The following day, transfected cells were seeded in 6-well dishes. At each indicated timepoint post-seeding, cells were harvested with trypsin, stained with Trypan Blue and counted using a Countess II Automated Cell Counter. For viability analysis, each cell line was seeded in triplicate in a 96-well plate. Quantification was performed with a CyQuant XTT Cell Viability Assay kit (Invitrogen, X12223) according to manufacturer instructions. Following absorbance measurement, wells were replenished with fresh media and placed at 37°C with 5% CO_2_ until next timepoint.

### Statistical analyses

Statistical analyses were performed using GraphPad Prism 10 software. Statistical details including test used and dispersion measures are included in the respective figure legends. Significance values for all experiments were derived using a *P* value of 0.05 as cutoff. Representative experiments are shown in figure panels, unless otherwise indicated. At least 100 fibers were analyzed for DNA fiber analyses, 200 nuclei for quantitative imaging experiments and 120 comets for neutral comet assays. All experiments were performed at least twice.

## Results

### SNF2L promotes replication fork progression

**Figure 1.**
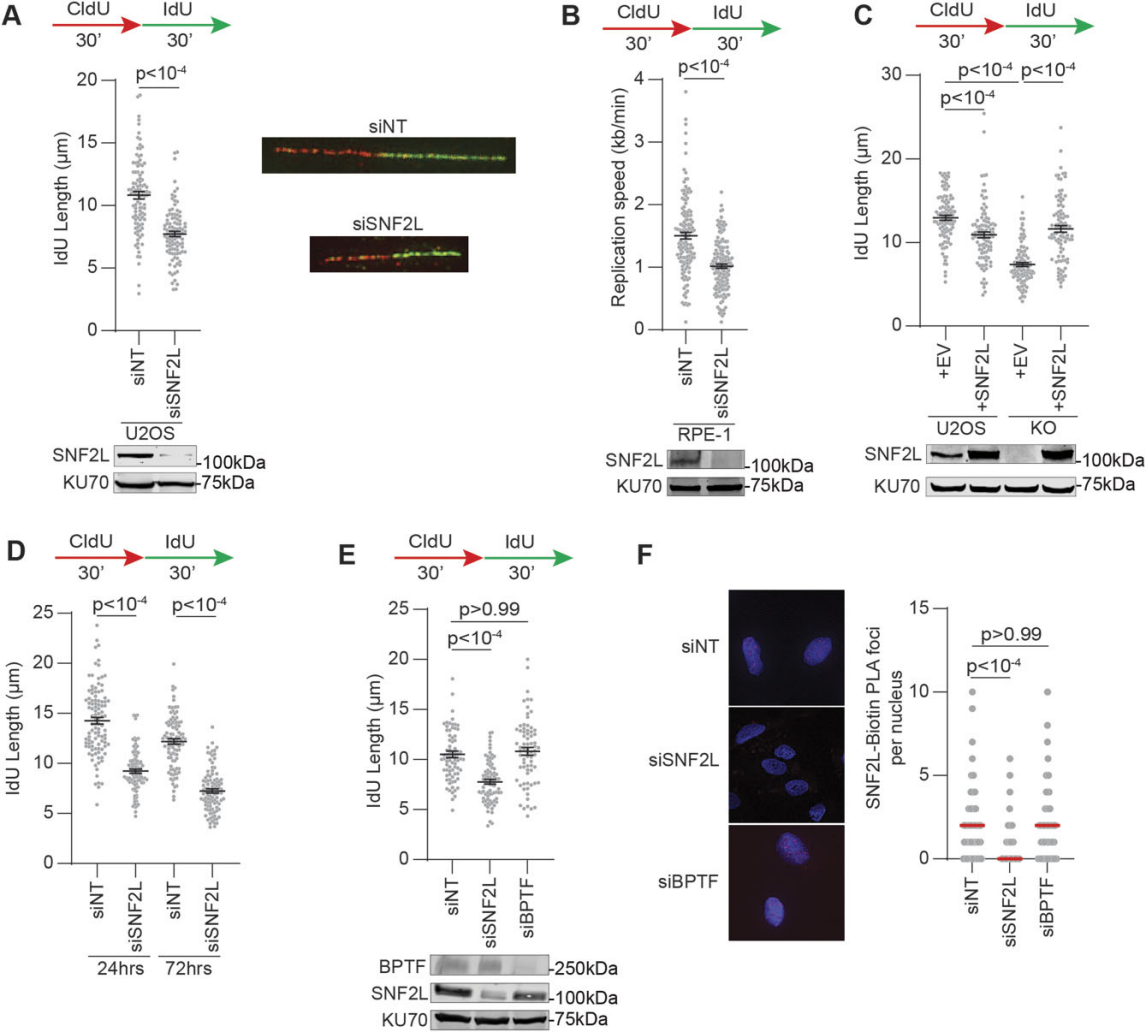
SNF2L loss perturbs replication fork progression. (**A** and **B**) Replication fork progression analyses were performed in U2OS cells by DNA spreading methodology (panel A) and hTERT-RPE-1 cells by DNA combing methodology (panel B). Immunoblots depict SNF2L knockdown using KU70 as loading control. Representative fiber images from U2OS cells are shown. (**C**) DNA fiber analysis was performed in parental U2OS and SNF2L KO cells that were transfected with either empty vector or cDNA expressing SNF2L. Immunoblot represents SNF2L expression prior to and after transfection with SNF2L cDNA. (**D**) siNT- and siSNF2L-transfected U2OS cells were subjected to DNA fiber analysis either 24 or 72 h post siRNA transfection. (**E**) siNT-, siSNF2L- and siBPTF-transfected U2OS cells were subjected to DNA fiber analysis as shown. Immunoblot depicts BPTF and SNF2L knockdown using KU70 as loading control. (**F**) SNF2L proximity to nascent DNA was detected using SIRF assay in the indicated siRNA-transfected U2OS cells using antibodies targeting SNF2L and biotinylated EdU. Black horizontal lines in panels A–E represent mean ± SEM. Red horizontal lines in panel F represent median. *P*-values were derived using Mann–Whitney test in panels A–B, and Kruskal–Wallis test with Dunn's multiple comparisons testing in panels C–F.

Replication fork proteomic analyses across several cell lines show chromatin remodeler SNF2L as a replisome component at unperturbed replication forks (29–31,56). Loss of SNF2L induces DNA damage during replication but the relevance of this is unclear (31). We performed single-molecule analyses of replicating DNA to investigate whether silencing SNF2L expression alters replication fork progression. Cells were sequentially pulsed with halogenated nucleoside analogs 5-chloro-2′-deoxyuridine (CldU) and 5-iodo-2′-deoxyuridine (IdU) for 30 min each and dual labeled replication tracts were quantified that represent actively elongating forks. SNF2L inactivation by siRNA transfection curtails fork speeds in U2OS cells (Figure 1A and Supplementary Figure S1A). Consistent with the identification of SNF2L at replication forks in hTERT-RPE-1 and HeLa cells, elongation rates are also curtailed when SNF2L is depleted in both cell types (Figure 1B and Supplementary Figure S1B) indicating that SNF2L function in replication fork progression is not cell type specific. To eliminate possible off target effects due to siRNA transfection, we generated SNF2L knockout (KO) clones in U2OS cells using CRISPR/Cas9 technology. Fork speeds were decreased across multiple isogenic KO clones (Supplementary Figure S1C). Furthermore, overexpressing catalytic isoform of SNF2L in KO cells restores fork speeds to near control levels (Figure 1C). We noticed that SNF2L cDNA expression in parental U2OS cells modestly affects replication elongation suggesting that SNF2L abundance at forks is modulated in cells. SNF2L presence at replication forks was also confirmed by SIRF assay (Figure 1F). Thus, SNF2L associates with active replication forks to facilitate fork progression.

To determine whether the observed fork progression defects in SNF2L-deficient cells are related to transcriptional changes, we compared fork speeds in cells transfected with siRNA targeting SNF2L 24 and 72 h post transfection. We observe that SNF2L depletion within 24 h is sufficient to slow fork elongation (Figure 1D and Supplementary Figure S1D) suggesting that alterations in fork speed are not due to large changes in transcription. SNF2L absence also minimally impacts proliferation (Supplementary Figure S1E and S1F) and cell cycle progression (Supplementary Figure S1G). To further support this observation, we transiently depleted NURF chromatin remodeling complex subunit BPTF (22). BPTF recognizes H3K4me3 to target SNF2L to promoter sites (57). We find that replication elongation is unaltered when BPTF is silenced (Figure 1E) and that SNF2L association with forks remains unperturbed when BPTF is depleted (Figure 1F). These results suggest that the reduced fork speed due to SNF2L absence is likely independent of transcriptional alterations.

### Forks are converted into DSBs when SNF2L is absent

**Figure 2.**
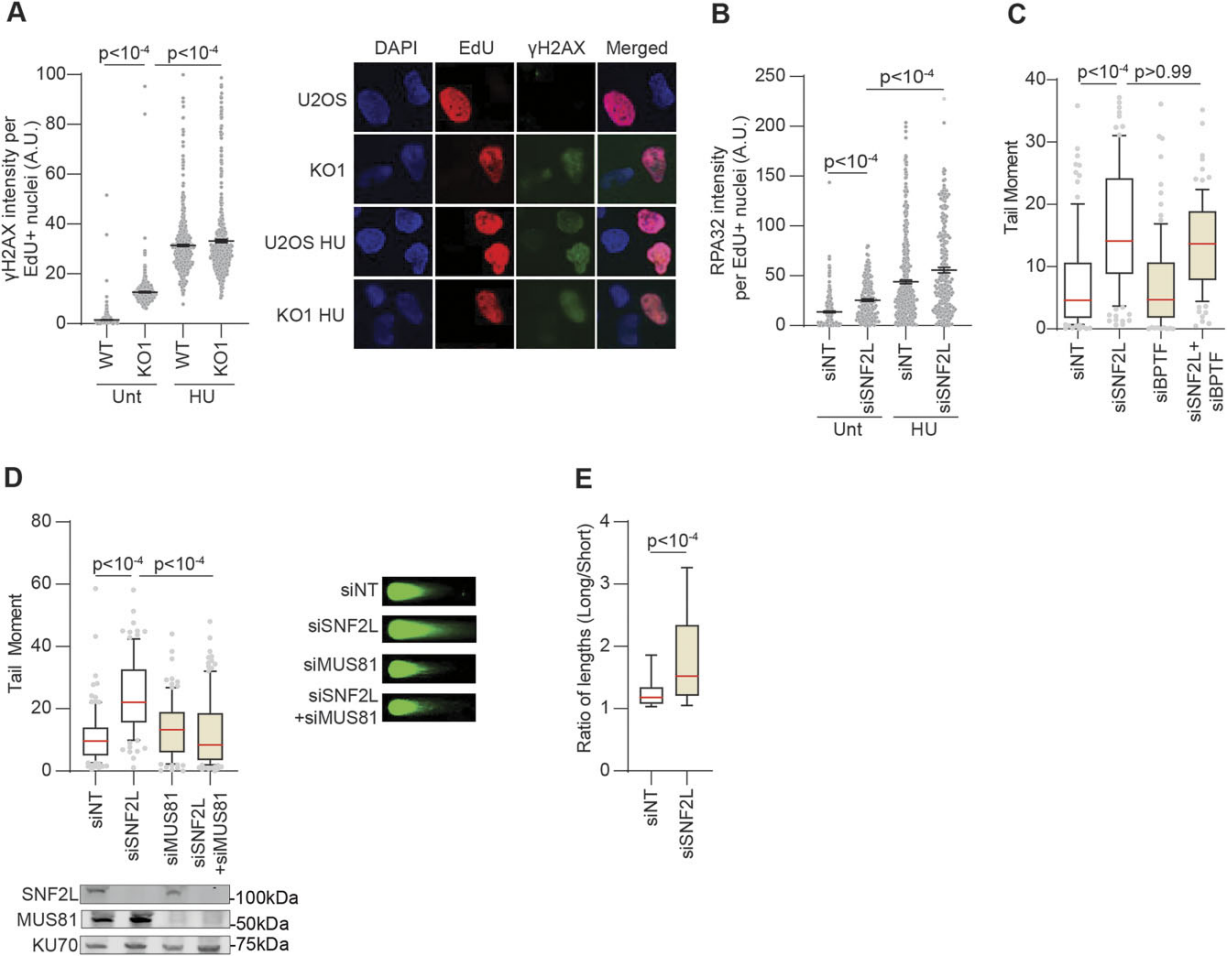
SNF2L prevents replication fork collapse into double strand breaks. (**A**) Parental U2OS and SNF2L KO1 cells were left either untreated or treated with HU for 4 h prior to immunostaining for DNA damage marker γH2AX. Representative immunofluorescence images of nuclei for the indicated samples are shown. (**B**) siNT- and siSNF2L-transfected U2OS cells were left either untreated or treated with HU for 4 h prior to immunostaining for RPA32. In both A and B, cells were pulse labeled with EdU for 10 min prior to HU exposure to identify replicating population. (**C**) Tail moments were quantified using neutral comet assay in U2OS cells transfected with the indicated siRNAs. (**D**) Tail moments were quantified using neutral comet assay in U2OS cells transfected with the indicated siRNAs. Immunoblot depicts knockdown of SNF2L and MUS81 using KU70 as loading control. Representative immunofluorescence images of comets for the indicated samples are shown. (**E**) Ratio of sister fork lengths was measured using DNA fiber analyses in U2OS cells transfected with the indicated siRNAs. Black horizontal lines in panels A and B represent mean ± SEM. Red horizontal lines in box and whisker plots in panels C, D and E represent median. *P*-values were derived using Kruskal–Wallis test with Dunn's multiple comparisons testing in panels A–D, and Mann–Whitney test in panel E. A.U.= arbitrary units.

Next, we tested whether impaired fork progression in SNF2L-deficient cells is associated with reduced stability of replication forks. In the absence of added replication stress, SNF2L knockdown increases levels of DNA damage marker γH2AX (Figure 2A) as well as chromatin-bound RPA32 in EdU-positive cells (Figure 2B). The levels of γH2AX and RPA32 in SNF2L-deficient conditions are further elevated in presence of high dose hydroxyurea (Figure 2A and B). Moreover, SNF2L-deficient cells exhibit elevated levels of DSBs as measured by neutral comet assay in conditions without exogenous genotoxic stress (Figure 2C). While BPTF-depleted cells do not exhibit accumulation of DSBs, loss of SNF2L and BPTF triggers break accumulation. This result suggests that, like replication fork progression, the function of SNF2L in preventing breaks is uncoupled from BPTF. The induction of DNA breaks in the absence of SNF2L is also observable in HEK293T and RPE-1 cells (Supplementary Figure S2A). SNF2L KO cells exhibit increased breaks that are rescued by complementation using SNF2L cDNA (Supplementary Figure S2B) and show increased G2/M DNA synthesis (Supplementary Figure S2C) reflecting the usage of salvage pathways to complete DNA replication after S-phase (58). MUS81 is a structure-specific endonuclease that cleaves stalled replication forks to resolve replication stress (39,59,60). Thus, we asked whether break accumulation in SNF2L-deficient cells is dependent on MUS81. We observe that DNA breaks in SNF2L-silenced cells are rescued when MUS81 is depleted (Figure 2D). These data indicate that loss of SNF2L causes MUS81-dependent cleavage of replication forks.

Since SNF2L absence curtails replication fork progression and concomitantly generates DSBs, we quantified the degree of replication fork asymmetry by DNA combing analyses to test whether forks undergo spontaneous collapse when SNF2L is deleted. For this purpose, we analyzed bidirectional replication forks and plotted the ratio of sister fork lengths. While the ratio of second-labelled tract lengths at bidirectional replication forks remains mostly unchanged in control cells, the frequency of asymmetric sister forks is elevated in SNF2L-delpeted cells (Figure 2E and Supplementary Figure S2D). Collectively, these data indicate that SNF2L prevents replication fork collapse into DSBs.

### Fork deceleration in SNF2L-deficient cells is dependent on replication fork reversal

**Figure 3.**
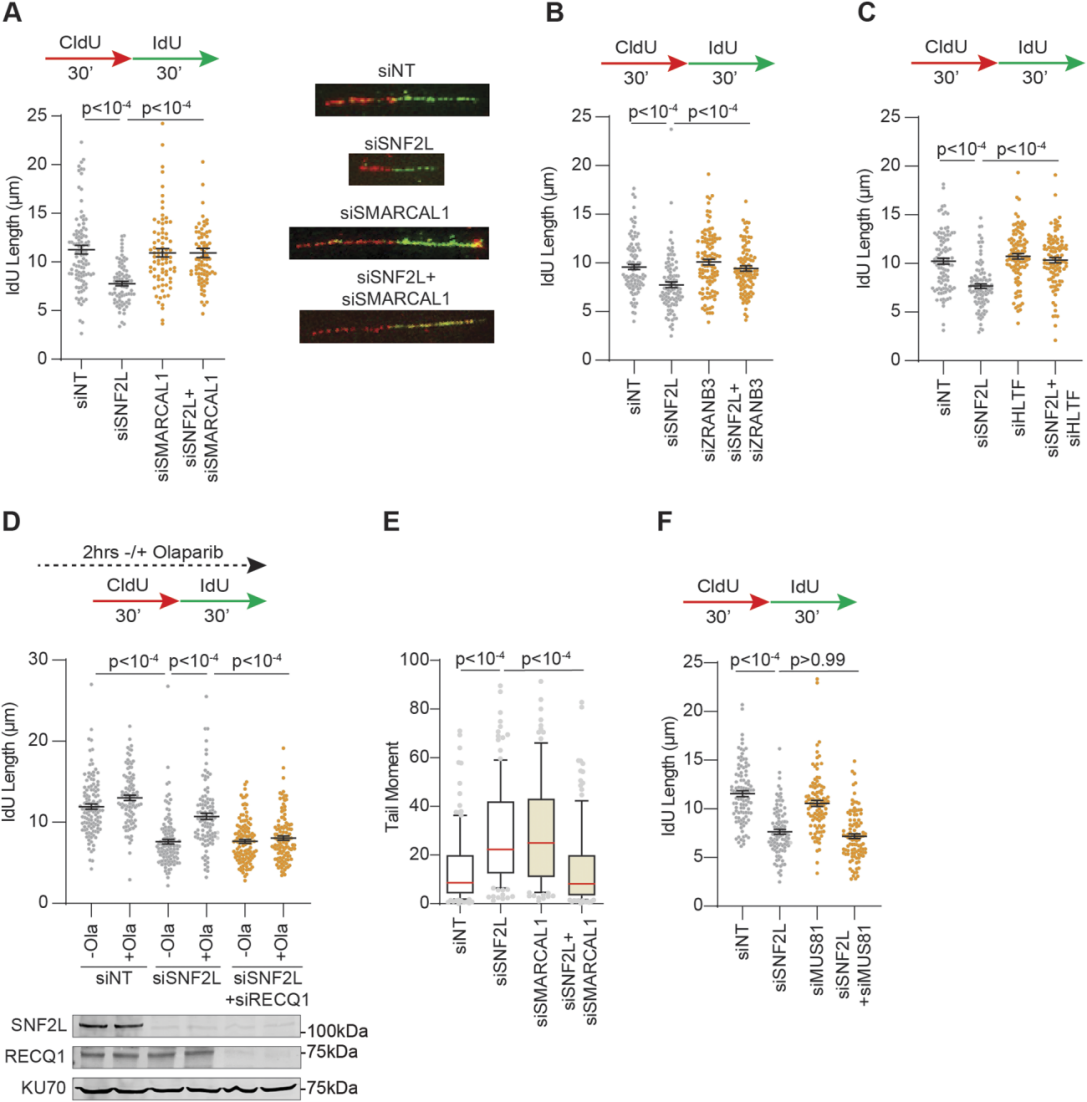
Fork reversal slows replication elongation in SNF2L-deficient conditions. (**A–C**) U2OS cells transfected with the indicated siRNAs were subjected to DNA fiber analysis to measure IdU lengths using dual labeled replication tracts. Representative fiber images for the indicated samples in panel A are shown. (**D**) U2OS cells transfected with the indicated siRNAs were subjected to DNA fiber analysis to measure IdU lengths using dual labeled replication tracts. Cells were left either untreated or pretreated with 10μM PARPi Olaparib for 2 h. Immunoblot depicts knockdown of SNF2L and RECQ1 using KU70 as loading control. (**E**) Tail moments were quantified using neutral comet assay in U2OS cells transfected with the indicated siRNAs. (**F**) U2OS cells transfected with the indicated siRNAs were subjected to DNA fiber analysis to measure IdU lengths using dual labeled replication tracts. Black horizontal lines in panels A–D and F represent mean ± SEM. Red horizontal lines in box and whisker plots in panel E represent median. *P*-values were derived using Kruskal–Wallis test with Dunn's multiple comparisons testing in all panels.

MUS81 cleaves multiple DNA substrates, including reversed replication forks that resemble four-way Holliday junctions (39). The MUS81-dependent DSB occurrence with slowed elongation in SNF2L-deficient cells prompted us to consider the possibility of spontaneous fork reversal events in the absence of SNF2L. Unwanted replication fork reversal can be problematic for otherwise unperturbed forks causing impaired fork progression and subsequent collapse into DSBs (36,61). We first asked whether decreased fork speed in SNF2L-deficient cells is dependent on fork reversal enzymes. Inactivating DNA translocase SMARCAL1 by siRNA-mediated knockdown restores fork elongation rates in SNF2L-depleted cells (Figure 3A and Supplementary Figure S3A). Replication speed in SNF2L-deficient cells is also rescued by depleting either ZRANB3 or HLTF (Figure 3B, C and Supplementary Figure S3A) consistent with their cooperative nature in regulating replication fork reversal (33). Similar dependency on fork remodelers SMARCAL1 and HLTF is also observed in SNF2L KO cells (Supplementary Figure S3B, S3C and S3D) suggesting that fork reversal is responsible for slowing elongation in SNF2L-deficient conditions. To further corroborate these observations, we treated SNF2L-deficient cells with PARP inhibitor Olaparib. Olaparib blocks PARP-dependent fork reversal and aids replication fork restart through RECQ1 (62). Acute exposure to PARP inhibitor restores fork elongation rate in SNF2L-deficient cells (Figure 3D). This rescue is abolished upon transient depletion of RECQ1 helicase (Figure 3D) indicating that SNF2L inactivation causes unfavorable remodeling of replication forks and thereby provides a replication intermediate for MUS81-dependent DSB induction. To test this prediction, we asked whether DSB accumulation in SNF2L-depleted cells is dependent on fork reversal enzyme SMARCAL1. Depleting SMARCAL1 abrogates break accumulation in SNF2L-depleted cells (Figure 3E) and three independent SNF2L KO clones (Supplementary Figure S3E) indicating that SNF2L prevents fork reversal-dependent collapse into DSBs. While MUS81 depletion rescues DNA breaks, it is unable to restore fork progression in SNF2L-deficient cells (Figure 3F). We conclude that indiscriminate replication fork remodeling in the absence of SNF2L slows DNA synthesis rates and consequentially drives MUS81-dependent replication fork collapse.

### SNF2L suppresses formation of replication gaps

**Figure 4.**
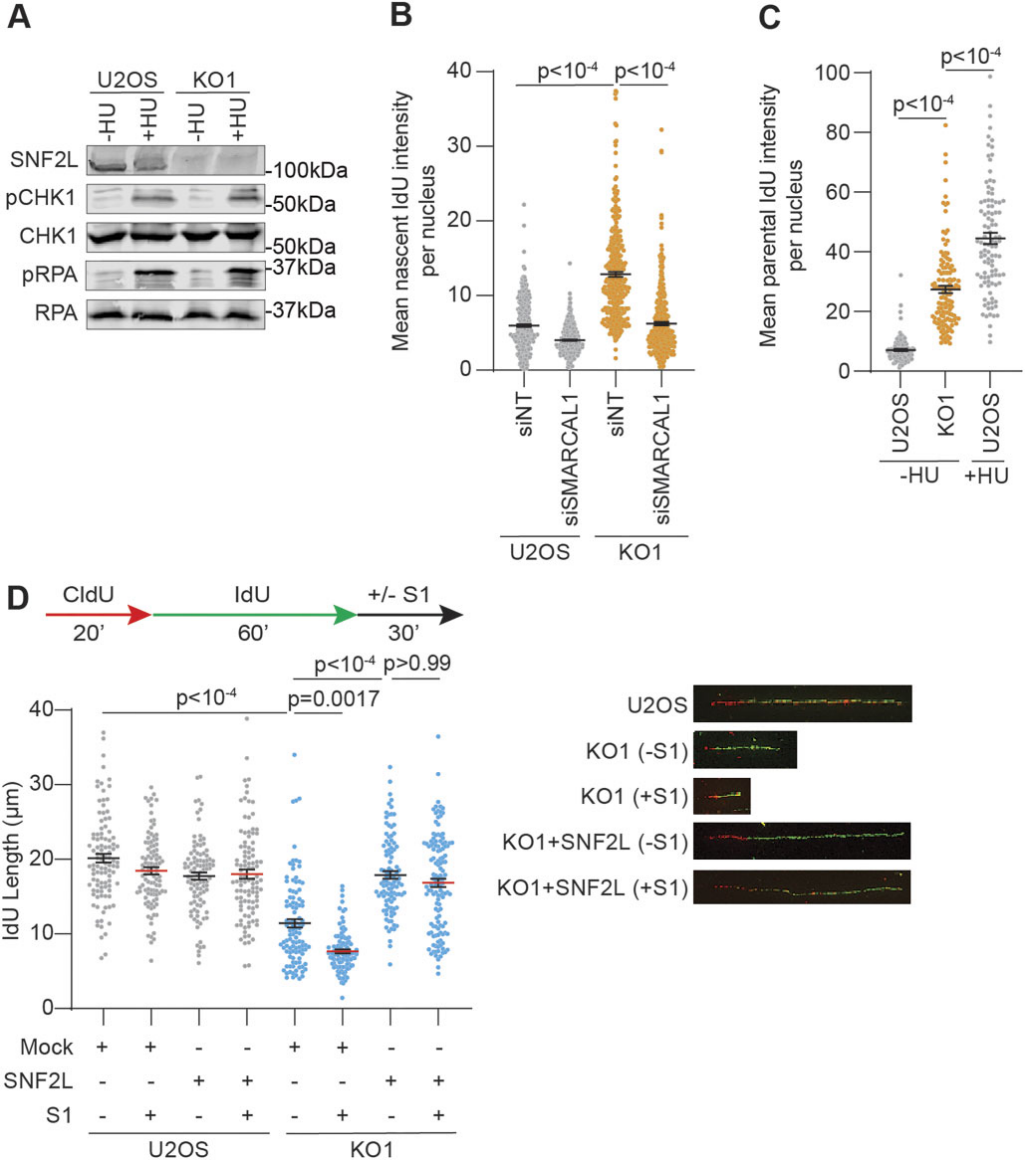
Loss of SNF2L induces nascent strand gap formation. (**A**) Parental U2OS and SNF2L KO1 cells were either left untreated or treated with 4 mM HU for 4 h. Cellular extracts were analyzed for ATR induction by immunoblotting. RPA was used as a loading control. (**B**) Parental U2OS and SNF2L KO1 cells transfected with the indicated siRNAs were analyzed for exposed nascent IdU in native conditions. (**C**) Parental U2OS and SNF2L KO1 cells were analyzed for exposed parental IdU in native conditions. HU-treated siNT cells were used as positive control. (**D**) To detect gaps in nascent DNA, mock or SNF2L cDNA transfected parental U2OS and SNF2L KO1 cells were subjected to DNA fiber analysis with or without S1 nuclease as per schematic. IdU lengths were measured using dual labeled replication tracts. Representative fiber images for the indicated samples are shown. Horizontal lines in panels B–D represent mean ± SEM. *P*-values were derived using Kruskal Wallis test with Dunn's multiple comparisons testing in panels B–D.

In unchallenged conditions, SNF2L-deficient cells exhibit elevated levels of chromatin-bound RPA32 (Figure 2B) indicating increased prevalence of exposed single-stranded DNA (ssDNA) at replication forks. Unlike high dose hydroxyurea (HU) that induces ATR activation by persistent stalling of forks, SNF2L-deleted cells show minimal activation of ATR checkpoint signaling as assessed by probing for ATR targets CHK1 (S317) and RPA32 (S33) (Figure 4A). The observed lack of ATR signaling is not due to defects in ATR activation since robust CHK1 and RPA32 phosphorylation is observed when SNF2L KO cells are treated with 4 mM HU (Figure 4A). This suggests that replication forks in SNF2L absence are differentially processed compared to HU-treated cells. To further characterize the source of exposed ssDNA, we considered two possibilities- conversion of nascent DNA to ssDNA and appearance of increased parental ssDNA (36). To measure exposed nascent ssDNA, we pulse labeled cells with 10 min of nucleoside analog IdU to measure nascent IdU nuclear intensities using non-denaturing conditions. Quantitative imaging indicates that nascent DNA converts to ssDNA more frequently in SNF2L-deficient conditions compared to SNF2L-proficient conditions (Figure 4B and Supplementary Figure S4A). Despite slower fork progression rates, SNF2L-deficient cells exhibit increased nascent ssDNA which may represent regressed arms of replication forks when SNF2L is lost (36). Given fork remodeling slows replication elongation rates in cells without SNF2L, we asked whether increased presence of nascent ssDNA is dependent on replication fork reversal. Silencing SMARCAL1 decreases nascent ssDNA signal in SNF2L-depleted cells (Figure 4B and Supplementary Figure S4A), while MUS81 depletion does not (Supplementary Figure S4B), indicating that exposed nascent ssDNA primarily arises from fork remodeling events when SNF2L is absent.

To measure presence of exposed parental ssDNA, cells were labeled with IdU overnight followed by release into IdU-free fresh media for 2 h and imaged using non-denaturing conditions. Expectedly, 2 h of HU treatment causes an increase in parental ssDNA compared to untreated control cells. In comparison, SNF2L-deficient cells also exhibit increased parental ssDNA but to a lesser extent relative to HU-treated conditions (Figure 4C). Unlike HU-treated cells that generate uncoupled replication forks and subsequent activation of ATR signaling cascade (63), unchallenged SNF2L-silenced cells lack significant induction in ATR signaling. These results prompted us to examine replication-associated gaps as a likely source for exposed parental ssDNA in SNF2L-deficient conditions. To test this, we adopted a modified version of DNA fiber analysis that incorporates 30 min of S1 nuclease after labeling cells with the second thymidine analog (54). Unfilled ssDNA gaps in the second label will be cleaved by S1 nuclease resulting in shortened IdU lengths. While IdU lengths are minimally sensitive to S1 nuclease in parental U2OS cells, IdU lengths in SNF2L KO cells are shortened after treatment with S1 nuclease indicating accumulation of ssDNA gaps on newly synthesized DNA (Figure 4D). Shortened nascent DNA tracts are also sensitive to S1 nuclease in HeLa (Supplementary Figure S4C) and hTERT-RPE1 cells (Supplementary Figure S4D) when SNF2L is removed. Since DNA fiber analyses cannot discriminate leading strand from the lagging strand, the observed shortening of nascent DNA tracts by S1 nuclease indicates that gap accumulation occurs on both sister chromatids. Importantly, complementation of SNF2L KO cells in U2OS with SNF2L cDNA abrogates sensitivity to S1 nuclease demonstrating that SNF2L functions to suppress ssDNA replication gaps (Figure 4D). We also tested whether depletion of SNF2H, a closely related paralog of SNF2L, generates replication gaps. Downregulating SNF2H slows fork speeds (Supplementary Figure S4E). However, this is not due to accumulation of replication gaps. Additionally, ssDNA gaps continue to persist in SNF2L KO cells when SNF2H is depleted suggesting that SNF2L and SNF2H have non-redundant functions during DNA replication.

### MRE11 and EXO1 generate replication gaps in SNF2L-deficient cells

**Figure 5.**
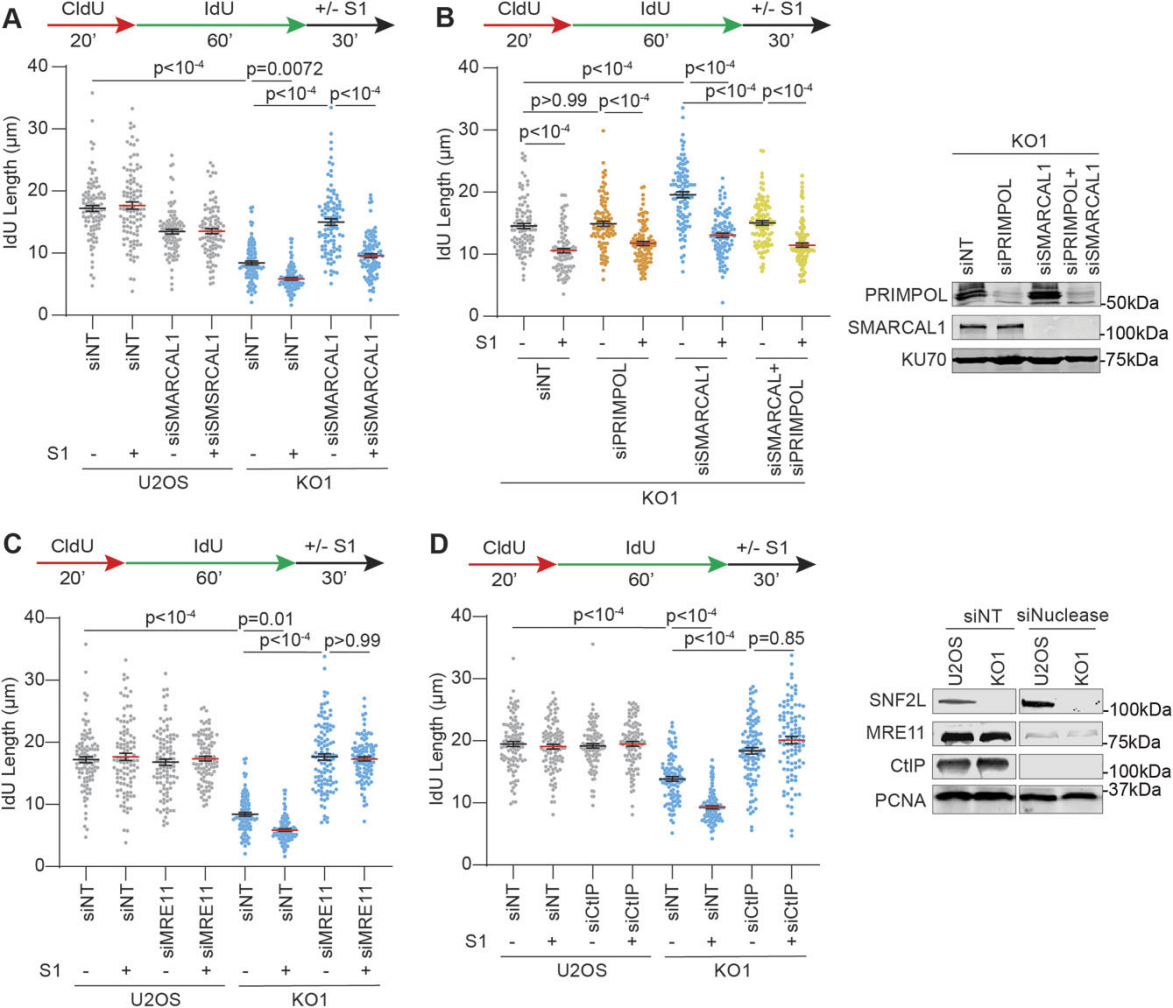
SNF2L prevents MRE11- and EXO1-dependent gap accumulation. (**A–D**) To detect gaps in nascent DNA, parental U2OS or SNF2L KO1 cells were transfected with the indicated siRNAs and subjected to DNA fiber analysis with or without S1 nuclease as per schematic. IdU lengths were measured using dual labeled replication tracts. Immunoblots depict knockdown efficiencies using either KU70 or PCNA as loading control. Horizontal lines in panels A–D represent mean ± SEM. *P*-values were derived using Kruskal Wallis test with Dunn's multiple comparisons testing in all panels.

Previous work has demonstrated that SMARCAL1 triggers replication fork reversal in response to gaps arising at fork junctions (64). Since gap accumulation in SNF2L-deficient cells accompany increased fork remodeling events, we first asked whether generation of ssDNA gaps is dependent on SMARCAL1. SMARCAL1 inactivation rescues elongation rates in SNF2L-deficient cells (Figure 5A) but this rescue does not abrogate gap accumulation indicating that ssDNA gaps continue to accumulate in SNF2L-deficient cells when fork reversal is inhibited. Indeed, exposure of parental DNA is amplified when SMARCAL1 is depleted (Supplementary Figure S5A). Additionally, reduced elongation rates and S1-nuclease sensitivity in SNF2L-deficient conditions is not rescued in FBH1-depleted cells indicating that FBH1-mediated replication fork remodeling is not involved in gap induction and slowed fork progression when SNF2L is absent (Supplementary Figure S5B). Suppressing replication fork reversal stimulates PRIMPOL-dependent repriming that results in accumulation of ssDNA gaps at replication forks (48,50). Thus, we tested whether the observed rescue in fork speed and concurrent presence of ssDNA gaps when SMARCAL1 is removed in SNF2L-deficient cells is dependent on PRIMPOL. In SMARCAL1-proficient conditions, siRNA-mediated depletion of PRIMPOL does not rescue fork elongation and S1-nuclease sensitivity (Supplementary Figure S5C) indicating that loss of SNF2L does not engage PRIMPOL-mediated repriming and subsequent DNA synthesis. In contrast, restoration of fork elongation in SMARCAL1-deficient, SNF2L KO cells is suppressed when PRIMPOL is absent (Figure 5B). Interestingly, nascent DNA in SMARCAL1- and PRIMPOL-deficient SNF2L KO cells remains sensitive to S1 nuclease which likely represents an alternate mechanism for nascent DNA gap synthesis. We conclude that increased fork remodeling in SNF2L-deficient cells arises due to gap-associated fork stalling events, and that ssDNA gap synthesis is not a direct consequence of PRIMPOL function unless fork reversal is removed.

Since gap accumulation in SNF2L-deficient cells is largely independent of PRIMPOL, we asked whether the incurrence of gaps is mediated by nucleases. To test this question, we investigated the dependency on MRE11, DNA2 and EXO1 that function in processing of nascent DNA at stalled replication forks (65). siRNA-mediated downregulation of either MRE11 or EXO1 restores fork elongation in SNF2L KO cells (Figures 5C and Supplementary Figure S5D). Moreover, nascent DNA in MRE11- or EXO1-depleted SNF2L KO cells show resistance to S1 nuclease indicating that gap synthesis and subsequent fork deceleration in SNF2L-deleted cells is dependent on MRE11 and EXO1. To test dependency on DNA2, and to determine whether the nuclease activity of MRE11 is responsible for gap accumulation, we utilized MRE11 nuclease inhibitor mirin and DNA2 nuclease inhibitor C5. While mirin exposure rescues fork speed and abrogates S1 nuclease sensitivity similar to MRE11-depletion, shortened nascent DNA molecules in C5-treated KO cells display S1 sensitivity (Supplementary Figure S5E) indicating that gap accumulation in SNF2L-deficient conditions is dependent on MRE11 nuclease. Mirin also rescues nascent ssDNA exposure in SNF2L-deficient conditions suggesting that MRE11 and EXO1 induce replication gaps to trigger fork reversal dependent nascent DNA exposure (Supplementary Figure S5F). CtIP is a co-factor for MRE11 endonuclease activity that helps initiate DNA end resection in a bi-directional manner (66). Intriguingly, CtIP removal abrogates S1 nuclease sensitivity and restores IdU lengths to near control levels indicating a role of MRE11 endonuclease in gap formation (Figure 5D). S1 fiber assays in an additional SNF2L KO clone show that gaps are driven by MRE11 and CtIP, but do not involve SMARCAL1 and PRIMPOL (Supplementary Figures S5G, S5H and S5I). As gap synthesis is independent of PRIMPOL, we infer that nascent DNA molecules are cleaved by MRE11 endonuclease to initiate gaps in SNF2L-deficient cells. These gaps are further expanded by exonuclease activities of MRE11 and EXO1, reminiscent of processing of gaps that arise via PRIMPOL-dependent repriming at stalled replication forks (46,47).

The abrogation of S1 nuclease sensitivity observed in DNA fiber analyses could be due to alleviation of gaps that occur in a strand-specific manner. Thus, we performed S1 nuclease assays on fibers prepared by DNA combing methodology which incorporates proteinase K treatment step that helps resolves sister chromatids (67). S1 nuclease assays with DNA combing indicate that depletion of EXO1, or MRE11 inhibition by either mirin or PFM-01, rescues sensitivity of nascent DNA molecules to S1 nuclease in SNF2L KO cells (Supplementary Figure S5J). We also tested whether Okazaki fragment maturation is defective in SNF2L-deficient cells by assessing PAR intensity in S-phase nuclei. Defects in lagging strand synthesis engage PARP which is detectable using short exposure to PARGi to block PAR turnover (68). While parental U2OS cells exhibit elevated PAR intensity in presence of PARGi, SNF2L KO cells show modest increase in S-phase PAR levels by quantitative imaging analyses (Supplementary Figure S5K). Coupled with the observations from DNA combing experiments, we conclude that MRE11 and EXO1 drive gap accumulation on both leading and lagging strands when SNF2L is absent.

### SNF2L organizes newly deposited chromatin to prevent gap accumulation

**Figure 6.**
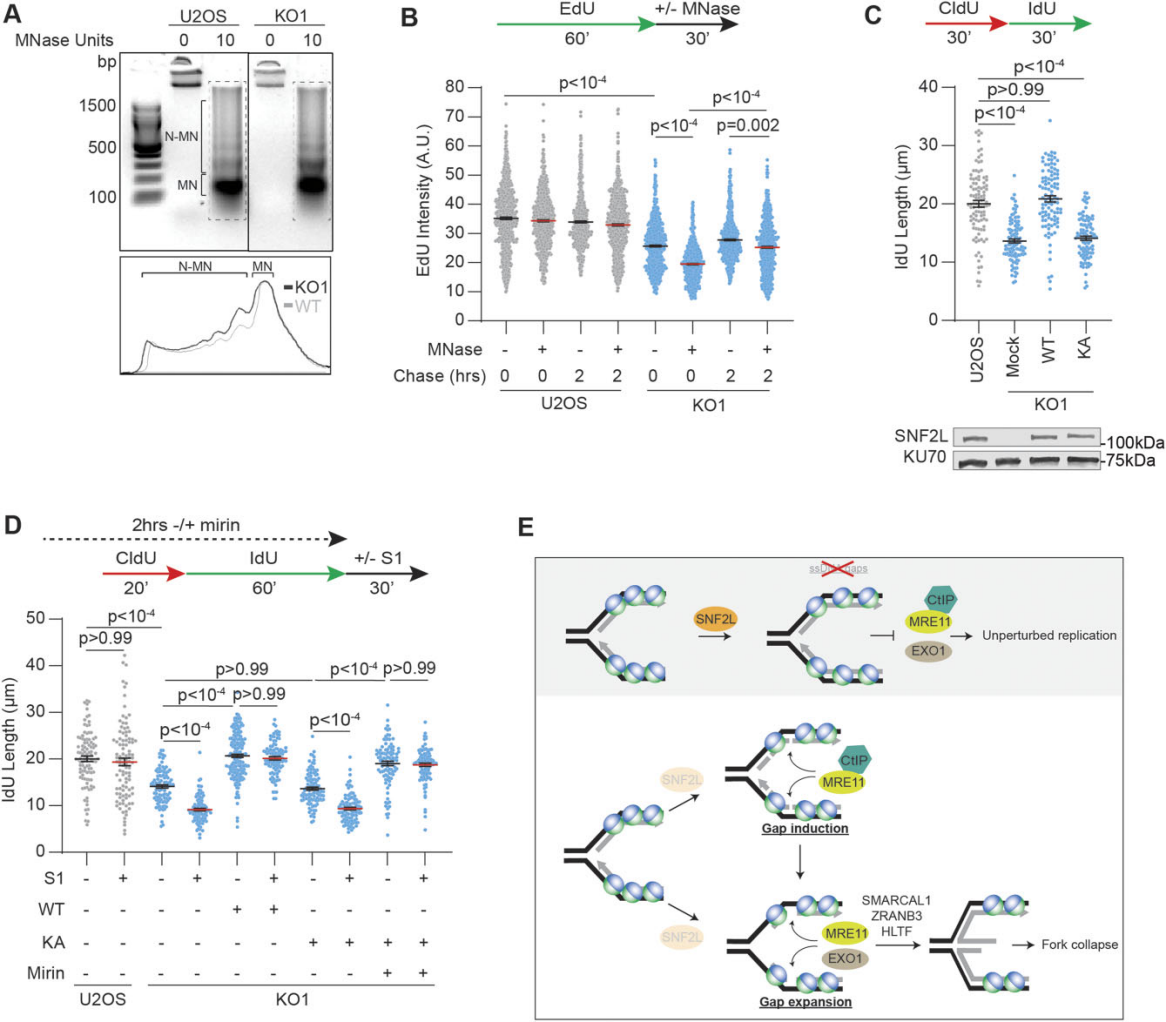
Defective chromatin organization causes gaps on nascent DNA. (**A**) Nucleosome banding patterns were analyzed in parental U2OS and SNF2L KO1 cells using MNase assay. The graph in the bottom panel depicts intensity distributions obtained from the dotted boxes as depicted. MN = mononucleosome, N-MN = non-mononucleosome. (**B**) Parental U2OS and SNF2L KO1 cells were pulsed with EdU for 60 min and permeabilized for IF analyses either immediately or after chasing into thymidine for 2 h. Cells were either untreated or treated with MNase for 30 min prior to fixation. EdU intensities were measured by quantitative imaging. A.U.= Arbitrary Units (**C**) SNF2L KO1 cells were transfected with empty vector, SNF2L-WT or SNF2L-K214A cDNA and subjected to DNA fiber analysis to measure IdU lengths using dual labeled replication tracts. Parental U2OS cells were included for comparison. Immunoblot validating SNF2L expression is depicted using KU70 as loading control. (**D**) Parental U2OS cells and SNF2L KO1 cells transfected with either SNF2L-WT or SNF2L-K214A cDNA were subjected to DNA fiber analysis with or without S1 nuclease as per schematic. IdU lengths were measured using dual labeled replication tracts. Where indicated, cells were pre-treated with mirin for 2 h. Horizontal lines in panels B-D represent mean ± SEM. p-values were derived using Kruskal Wallis test with Dunn's multiple comparisons testing in all panels. (**E**) Model- SNF2L travels with replication forks and organizes newly assembled nucleosomes to promote replication fork progression. Defective nascent chromatin assembly due to SNF2L absence causes gap accumulation resulting in fork collapse by fork reversal enzymes. The genesis of replication gaps occurs either by endonucleolytic resection by MRE11 or by an alternate mechanism. The subsequent gap accumulation is driven by MRE11 and EXO1.

SNF2L acts on nucleosomes to generate ordered arrays (69,70). *In vitro* studies show that MRX complex in yeast can nick DNA internally to the nucleosome to initiate gap synthesis (71). Thus, we hypothesized that SNF2L organizes nucleosomes behind replication forks to prevent aberrant nascent DNA gap accumulation. To test this prediction, we analyzed the pattern of nucleosomal distribution in parental and SNF2L KO U2OS cells using micrococcal nuclease (MNase) assay. We observe that chromatin is more accessible to MNase in KO cells which exhibit increased prevalence of non-mononucleosomal DNA fragments, consistent with altered chromatin compaction in SNF2L-depleted cells (Figure 6A). To determine whether the increased chromatin accessibility in SNF2L deficiency is distinct to nascent DNA compared to bulk chromatin, we assessed sensitivity of EdU labeled DNA to MNase by quantitative immunofluorescence analyses. Cells were either pulse labeled with EdU for 1 h or pulsed with EdU followed by chase into thymidine for 2 h following which MNase was added to digest chromatin. Nuclear intensities in parental U2OS cells remain mostly unaffected indicating marginal sensitivity to MNase (Figure 6B). Expectedly, SNF2L KO cells exhibit reduced EdU intensity due to slow fork progression rates. After subjecting to MNase treatment, the reduction in EdU intensity is exacerbated demonstrating that nascent chromatin is more accessible to MNase when SNF2L is lost. Furthermore, EdU-labeled DNA that is chased into mature chromatin exhibits reduced MNase sensitivity indicating that heightened MNase sensitivity is largely restricted to nascent chromatin in SNF2L-deficient cells. Since silencing SNF2L induces gaps in nascent DNA, we tested the link between chromatin accessibility and ssDNA gap synthesis by performing a pulse chase experiment using S1 nuclease assay. While newly synthesized DNA in SNF2L KO cells is sensitive to S1 nuclease, the observed S1 sensitivity gradually diminishes when the nascent DNA is chased into mature chromatin (Supplementary Figure S6A). These data suggest that defects in chromatin assembly on newly synthesized DNA trigger gap accumulation.

To further attribute the observations to the nucleosome remodeling activity of SNF2L, we substituted a conserved lysine residue in the Walker A consensus motif GXXGK(T/S) (Supplementary Figure S6B), responsible for binding and hydrolyzing ATP (72), to alanine (K214A, hence called KA). When KA mutant is expressed to similar levels as wild-type SNF2L, it fails to restore replication fork progression rates (Figure 6C) despite retaining the ability to localize to replication forks (Supplementary Figure S6C). Additionally, unlike wild-type SNF2L, re-expressing SNF2L-KA in SNF2L KO cells renders nascent DNA to be susceptible to MNase (Supplementary Figure S6D) suggesting that the ATPase-dependent nucleosome remodeling function is critical for proper chromatin assembly on nascent DNA. Since SNF2L loss triggers gap accumulation, we considered whether the chromatin remodeling activity of SNF2L also aids in suppressing nascent DNA gaps. To test this, we performed S1 nuclease assay in SNF2L KO cells expressing either wild-type SNF2L or KA mutant. Nascent DNA lengths restored by either transient or stable expression of wild-type SNF2L do not exhibit S1 nuclease sensitivity (Figure 6D and S6E). In comparison, nascent DNA in KA mutant expressing cells continue to accumulate ssDNA gaps as demonstrated by decrease in IdU lengths by S1 nuclease. Importantly, blocking the nuclease activity of MRE11 by mirin restores IdU lengths and prevents gap accumulation in KA mutant expressing cells (Figure 6D). Collectively, these results indicate that SNF2L-mediated chromatin assembly on nascent DNA prevents ssDNA gap accumulation.

## Discussion

ssDNA gaps are common replication intermediates whose resolution is vital for genome stability and chemotherapy response. ssDNA gaps arise during base excision repair (73), nucleotide excision repair (74), defective lagging strand maturation (44,75) and PRIMPOL-mediated repriming (32). Herein, we outline a mechanism of ssDNA gap generation when SNF2L is absent, implicating a role of chromatin remodeling in suppressing gap synthesis on nascent DNA molecules. We find that SNF2L travels with replication forks to prevent gap accumulation that is primarily triggered by nucleolytic resection. When SNF2L is silenced, ssDNA gaps cause replication fork remodeling by fork reversal enzymes resulting in fork collapse and DSB induction. We propose a model wherein SNF2L uses its catalytic activity to reorganize nucleosomes behind the replication fork to prevent aberrant cleavage of nascent DNA molecules by MRE11 and additional gap expansion by MRE11 and EXO1 (Figure 6E).

Newly synthesized DNA molecules are rapidly assembled into chromatin with the presence of nucleosomes in proximity to the fork junction (76,77). The process of chromatin maturation entails generating immature nucleosomes devoid of histone H1 followed by regularly assembled nucleosome arrays (78). Consistent with this model, newly replicated chromatin is less stable and more susceptible to nuclease-mediated digestion compared to bulk chromatin (79). Our data show that MNase sensitivity of nascent DNA in SNF2L-deficient cells is enhanced relative to bulk chromatin indicative of defective chromatin maturation behind the replication fork. The MNase susceptibility likely arises due to alterations in chromatin assembly or remodeling given nucleosomes are rapidly deposited and repositioned on newly synthesized DNA (80). Based on the evidence that SNF2L functions in nucleosomal spacing, we speculate that SNF2L remodels nucleosomes into highly organized arrays to prevent aberrant gap accumulation. ISWI containing ATP-utilizing chromatin assembly and remodeling factor (ACF) complex in Drosophila reassemble regularly spaced nucleosomes *in vitro* while yeast ISWI spaces nucleosomal arrays as part of ACF and CHRAC complexes (70,81). Moreover, both SNF2L and SNF2H form interchangeable complexes with regulatory subunits and exhibit increased ATP hydrolysis with chromatinized templates (24). Consistent with these observations, we find that the ability of SNF2L to mediate chromatin compaction on nascent DNA and suppress ssDNA gap accumulation is abolished when the SNF2L ATPase-dead mutant is expressed. Although ISWI chromatin remodelers are highly conserved from yeast to humans, the remodeling functions of ISWI vary depending on cellular and genomic context. As part of the NURF complex (22), SNF2L participates in regulating gene expression. In this regard, we find that depleting NURF-complex subunit BPTF does not mimic phenotypes of slow fork progression and S1-nuclease sensitivity observed in SNF2L-depleted cells indicating that the role of SNF2L in transcription and replication are uncoupled. Instead, the human paralog SNF2H was demonstrated to play a critical role in nucleosomal organization at CTCF sites of transcription (82). Intriguingly, NURF-complex subunits BPTF, RBBP7 and RBBP4 are also found to be enriched at unperturbed fork proteomes (29) suggesting that SNF2L travels with forks as part of the NURF complex. While BPTF recruits SNF2L to promoter regions (57), our results indicate that this dependency is not required for SNF2L-association with replication forks. Furthermore, SNF2H and SNF2L have disparate functions in DNA replication since we do not observe induction of ssDNA gaps when SNF2H is depleted. Our findings also show that SNF2L loss causes minimal defects in cell cycle progression and viability, which is likely attributed to gap resolution in G2/M phase of the cell cycle.

Our findings demonstrate that fork deceleration in SNF2L-deficient conditions is dependent on DNA translocases SMARCAL1, ZRANB3 and HLTF that trigger replication fork reversal (35). Acute exposure to PARPi, which blocks fork reversal and promotes RECQ1-dependent fork restart (62), restores fork speed indicating that replication fork reversal slows fork progression when SNF2L is absent. Removal of fork reversal enzyme SMARCAL1 also abrogates break induction in SNF2L-depleted cells supporting previous observations that unregulated fork reversal can trigger fork collapse and DNA break induction (36,61). Intriguingly, we find that nascent DNA molecules are also sensitive to S1 nuclease when SNF2L is removed which would suggest that replication fork reversal results in gap induction. Contrary to this prediction, we observe that gaps continue to persist when fork reversal is abolished indicating that gaps do not originate from fork reversal events. Furthermore, while gap accumulation in SNF2L-deficient conditions appears to be PRIMPOL-independent, removal of SMARCAL1 causes PRIMPOL-mediated gap synthesis and increase in lengths of nascent DNA tracts. Thus, our observations support a model whereby gaps arise on the replication forks and consequentially engage replication fork reversal to pause DNA synthesis. This model is consistent with fork reversal emanating as a response to replication gaps (83) and preferential engagement of fork reversal versus PRIMPOL mediated repriming in fork stalling conditions (48–50). Given that nascent DNA digestion is fork reversal dependent when forks are stalled with DNA lesions (65,84), we cannot exclude the possibility that MRE11 and EXO1 digest nascent DNA at reversed replication forks. However, we observe that blocking fork reversal enzymes sustains gap accumulation in a PRIMPOL-dependent manner arguing against MRE11 acting on regressed arms for gap synthesis.

Our studies indicate that nascent strand gap formation is driven by MRE11 and EXO1 nucleases but not DNA2. PRIMPOL-dependent gaps at stalled forks are processed by opposing resection directionalities—MRE11 in a 3′-5′ polarity and EXO1 in a 5′-3′ polarity (46,47). However, we find that gap synthesis in SNF2L-deficient cells is independent of PRIMPOL. Additionally, when fork reversal enzyme SMARCAL1 is depleted, fork speeds are restored in a PRIMPOL-dependent manner, but gaps continue to persist when both SMARCAL1 and PRIMPOL are depleted in SNF2L KO cells underlining an alternate source for gap synthesis. We postulate that cleavage of nascent DNA by MRE11 nuclease, in coordination with CtIP, is responsible for gap synthesis and provides entry points for additional processing by MRE11 and EXO1. In support of these observations, MRE11 can form gaps independently of PRIMPOL through its endonuclease activity (46). MRX-Sae2, yeast orthologs of MRE11 and CtIP, can act internally at nucleosomal-bound DNA indicating that presence of nucleosomes is not refractory to actions of MRE11 (71). In this model, MRX-Sae2 can endonucleolytically cleave 5′ strand of DNA at the nucleosomal periphery and chew back in a 3′-5′ direction generating a DNA gap which is further expanded by additional nuclease activities. Furthermore, this action occurs in an Sae2-dependent manner and supports our observations for CtIP-dependent gap induction in SNF2L-deficient cells. MRN can also freely diffuse along DNA that is coated with nucleosomes (85) demonstrating that nucleosomes are not obstacles to DNA recognition and scission by MRE11. We predict that SNF2L acts at replication forks to rapidly assemble and space the newly deposited nucleosomes to prevent aberrant DNA cleavage by MRN-CtIP which is a replisome component at actively elongating forks (29). Absence of SNF2L triggers MRE11 and EXO1 processing of nascent DNA resulting in heightened accumulation of replication gaps. The induction of gaps by MRE11 could occur either on linker DNA or nucleosomal DNA. Although nucleosomal DNA is refractory to MRE11-dependent cleavage (71,86), incomplete assembly of nucleosomes behind the replication fork could also enable MRE11 to cleave pre-nucleosomal DNA (87). An alternate scenario is induction of gaps that occur independently of MRE11 and processed by MRE11 and EXO1. In this context, repriming by pol alpha primase can provide gaps (47,88) on the lagging strand but not the leading strand. Future studies will be needed to investigate these possibilities.

## Supplementary Material

gkae903_Supplemental_File

## Data Availability

All data underlying this article will be shared by the corresponding author upon request.
