# Supplementary material for: SNF2L suppresses nascent DNA gap formation to promote DNA synthesis
Source: Nucleic Acids Res. 2024 Oct 16;52(21):13003–18. doi: 10.1093/nar/gkae903 (PMC11602140; doi:10.1093/nar/gkae903)
Supplement: gkae903_Supplemental_File [file gkae903_supplemental_file.pdf]

Figure S1

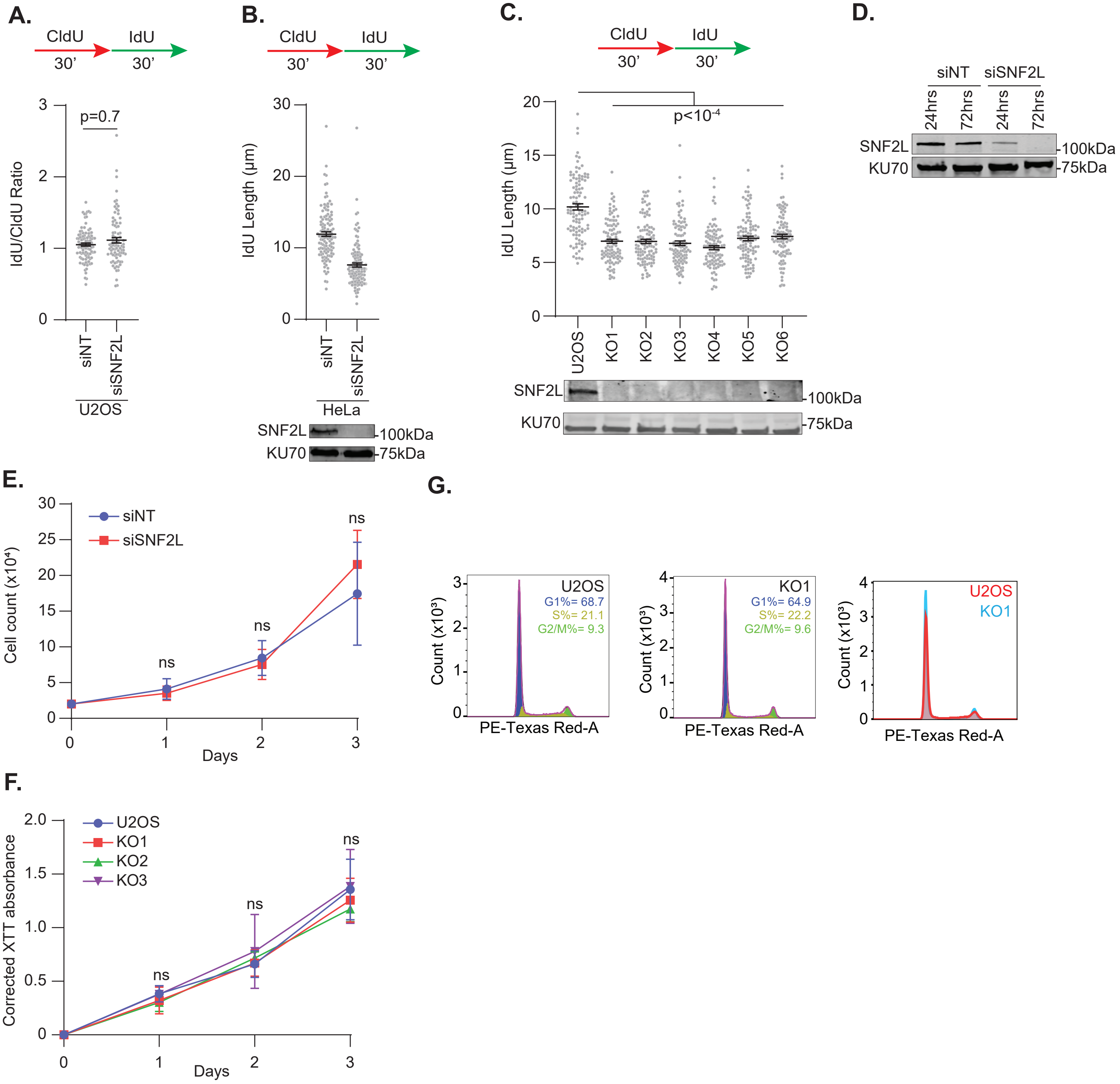

A.
CldU
IdU
30’
30’
p=0.7
IdU/CldU Ratio
siNT
siSNF2L
U2OS
B.
CldU
IdU
30’
30’
IdU Length (μm)
siNT
siSNF2L
HeLa
SNF2L
KU70
100kDa
75kDa
C.
CldU
IdU
30’
30’
p<10-4
IdU Length (μm)
U2OS
KO1
KO2
KO3
KO4
KO5
KO6
SNF2L
KU70
100kDa
75kDa
D.
siNT
siSNF2L
24hrs
72hrs
24hrs
72hrs
SNF2L
KU70
100kDa
75kDa
E.
siNT
siSNF2L
ns
ns
ns
Cell count (x10⁴)
Days
G.
U2OS
G1%= 68.7
S%= 21.1
G2/M%= 9.3
Count (x10³)
PE-Texas Red-A
KO1
G1%= 64.9
S%= 22.2
G2/M%= 9.6
Count (x10³)
PE-Texas Red-A
U2OS
KO1
Count (x10³)
PE-Texas Red-A
F.
U2OS
KO1
KO2
KO3
ns
ns
ns
Corrected XTT absorbance
Days

Figure S2

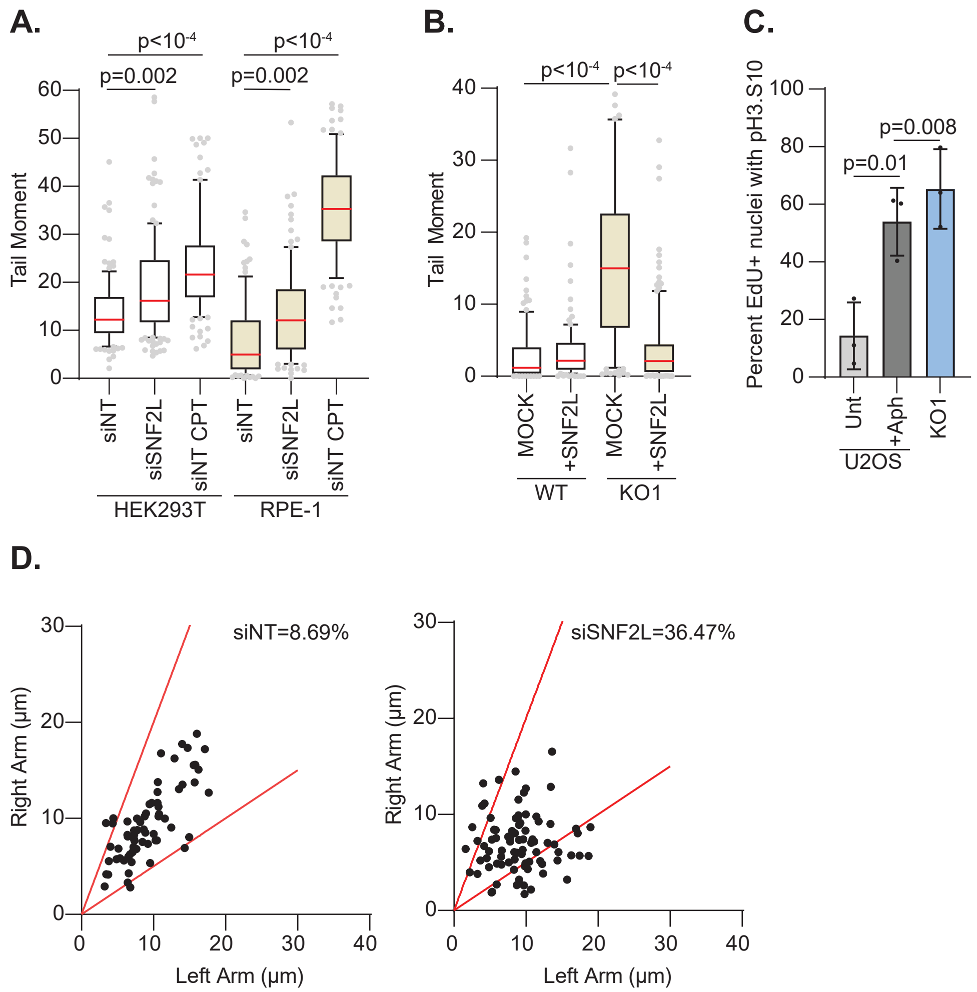
A.
p<10-4
p=0.002
p<10-4
p=0.002
Tail Moment
60
50
40
30
20
10
0
siNT
siSNF2L
siNT CPT
siNT
siSNF2L
siNT CPT
HEK293T
RPE-1
B.
p<10-4
p<10-4
Tail Moment
40
30
20
10
0
MOCK
+SNF2L
MOCK
+SNF2L
WT
KO1
C.
Percent EdU+ nuclei with pH3.S10
100
80
60
40
20
0
p=0.008
p=0.01
Unt
+Aph
KO1
U2OS
D.
siNT=8.69%
Right Arm (µm)
Left Arm (µm)
siSNF2L=36.47%
Right Arm (µm)
Left Arm (µm)

Figure S3

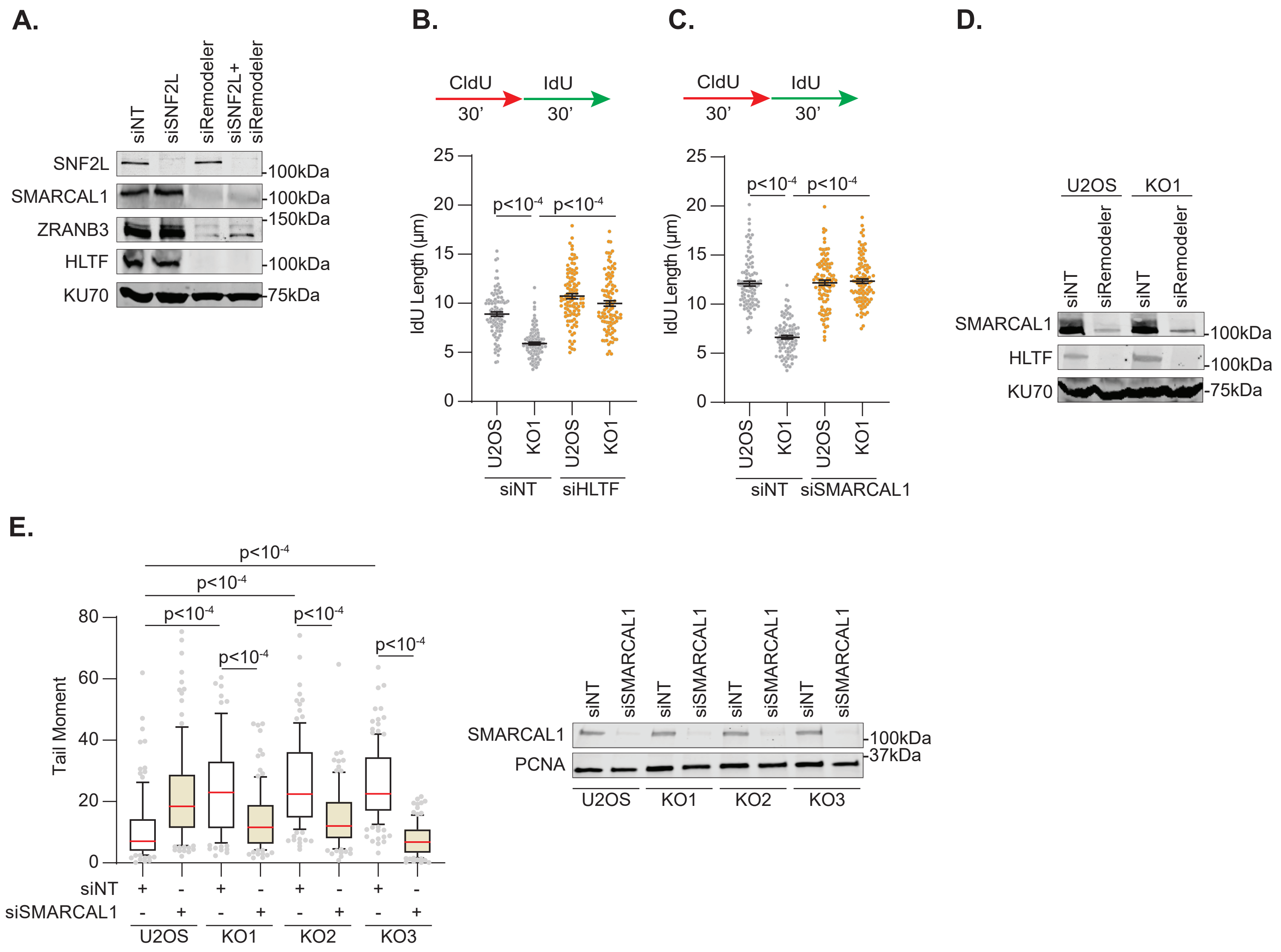

A.
siNT
siSNF2L
siRemodeler
siSNF2L+ siRemodeler
SNF2L
SMARCAL1
ZRANB3
HLTF
KU70
100kDa
100kDa
150kDa
100kDa
75kDa
B.
CldU
IdU
30'
30'
IdU Length (μm)
p<10-4
p<10-4
U2OS
KO1
U2OS
KO1
siNT
siHLTF
C.
CldU
IdU
30'
30'
IdU Length (μm)
p<10-4
p<10-4
U2OS
KO1
U2OS
KO1
siNT
siSMARCAL1
D.
U2OS
KO1
siNT
siRemodeler
siNT
siRemodeler
SMARCAL1
HLTF
KU70
100kDa
100kDa
75kDa
E.
Tail Moment
p<10-4
siNT
siSMARCAL1
U2OS
KO1
KO2
KO3
SMARCAL1
PCNA
100kDa
37kDa

Figure S4

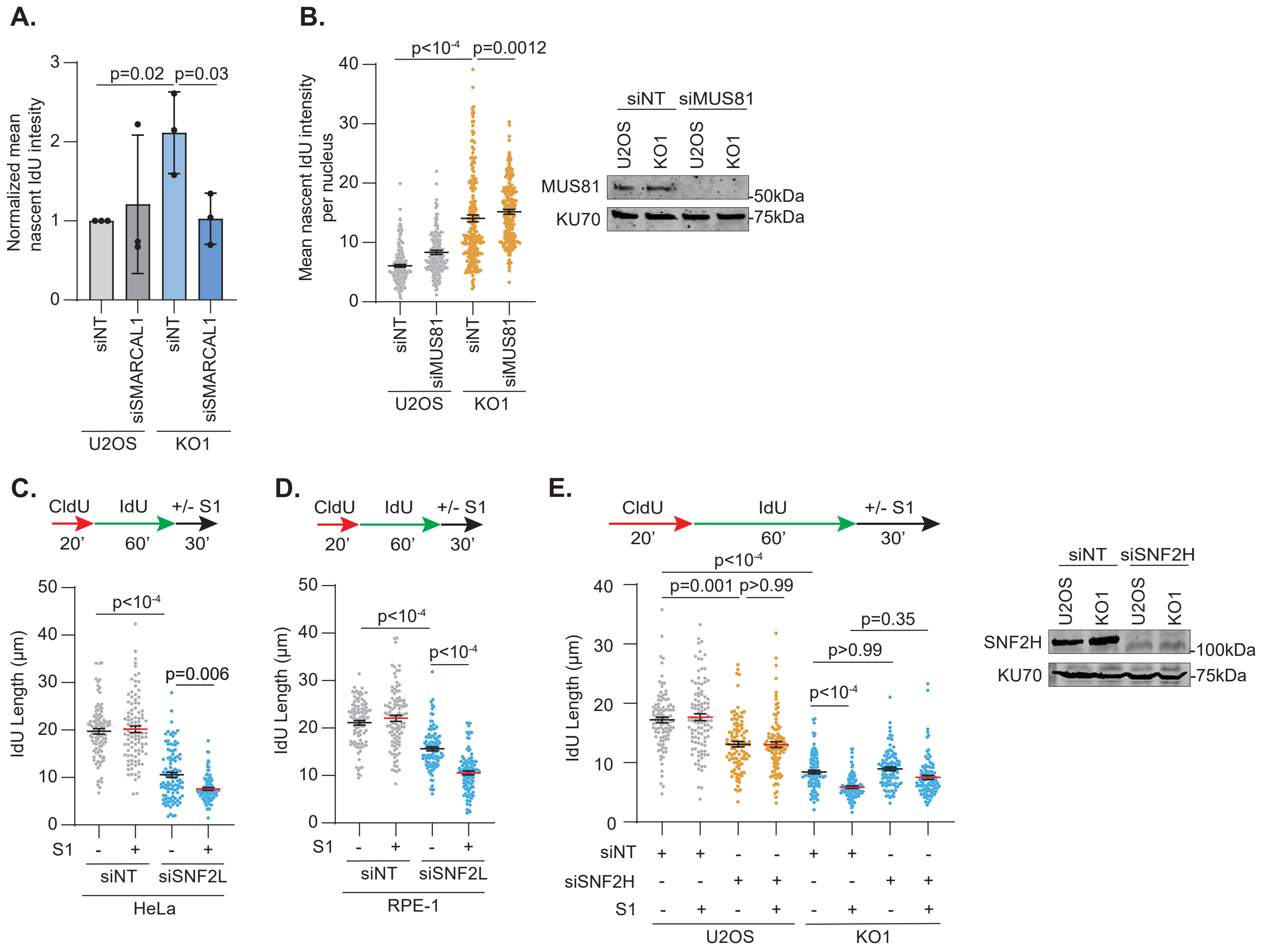

A.
Normalized mean nascent IdU intesity
p=0.02
p=0.03
siNT
siSMARCAL1
siNT
siSMARCAL1
U2OS
KO1
B.
Mean nascent IdU intensity per nucleus
p<10-4
p=0.0012
siNT
siMUS81
siNT
siMUS81
U2OS
KO1
siNT
siMUS81
U2OS
KO1
U2OS
KO1
MUS81
KU70
50kDa
75kDa
C.
CldU
IdU
+/- S1
20'
60'
30'
IdU Length (μm)
p<10-4
p=0.006
S1
siNT
siSNF2L
HeLa
D.
CldU
IdU
+/- S1
20'
60'
30'
IdU Length (μm)
p<10-4
p<10-4
S1
siNT
siSNF2L
RPE-1
E.
CldU
IdU
+/- S1
20'
60'
30'
IdU Length (μm)
p<10-4
p=0.001
p>0.99
p=0.35
p>0.99
p<10-4
siNT
siSNF2H
S1
U2OS
KO1
siNT
siSNF2H
U2OS
KO1
U2OS
KO1
SNF2H
KU70
100kDa
75kDa

Figure S5

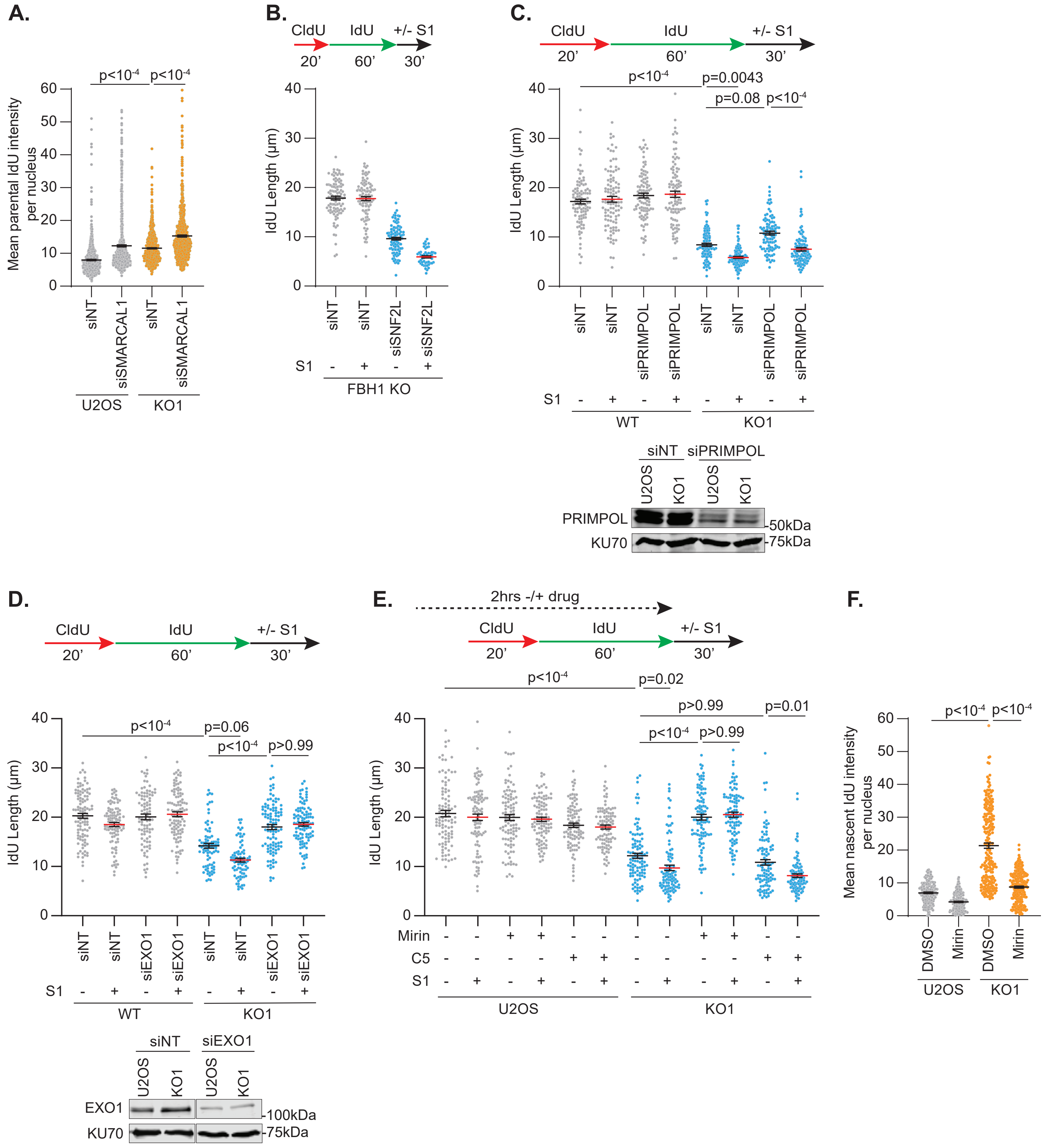

A.
p<10-4
p<10-4
Mean parental IdU intensity per nucleus
siNT
siSMARCAL1
siNT
siSMARCAL1
U2OS
KO1
B.
CldU
IdU
+/- S1
20’
60’
30’
IdU Length (µm)
siNT
siNT
siSNF2L
siSNF2L
S1
FBH1 KO
C.
CldU
IdU
+/- S1
20’
60’
30’
p<10-4
p=0.0043
p=0.08
p<10-4
IdU Length (µm)
siNT
siNT
siPRIMPOL
siPRIMPOL
siNT
siNT
siPRIMPOL
siPRIMPOL
S1
WT
KO1
siNT
siPRIMPOL
U2OS
KO1
U2OS
KO1
PRIMPOL
KU70
-50kDa
-75kDa
D.
CldU
IdU
+/- S1
20’
60’
30’
p<10-4
p=0.06
p<10-4
p>0.99
IdU Length (µm)
siNT
siNT
siEXO1
siEXO1
siNT
siNT
siEXO1
siEXO1
S1
WT
KO1
siNT
siEXO1
U2OS
KO1
U2OS
KO1
EXO1
KU70
-100kDa
-75kDa
E.
2hrs -/+ drug
CldU
IdU
+/- S1
20’
60’
30’
p<10-4
p=0.02
p>0.99
p=0.01
p<10-4
p>0.99
IdU Length (µm)
Mirin
C5
S1
U2OS
KO1
F.
p<10-4
p<10-4
Mean nascent IdU intensity per nucleus
DMSO
Mirin
DMSO
Mirin
U2OS
KO1

Figure S5

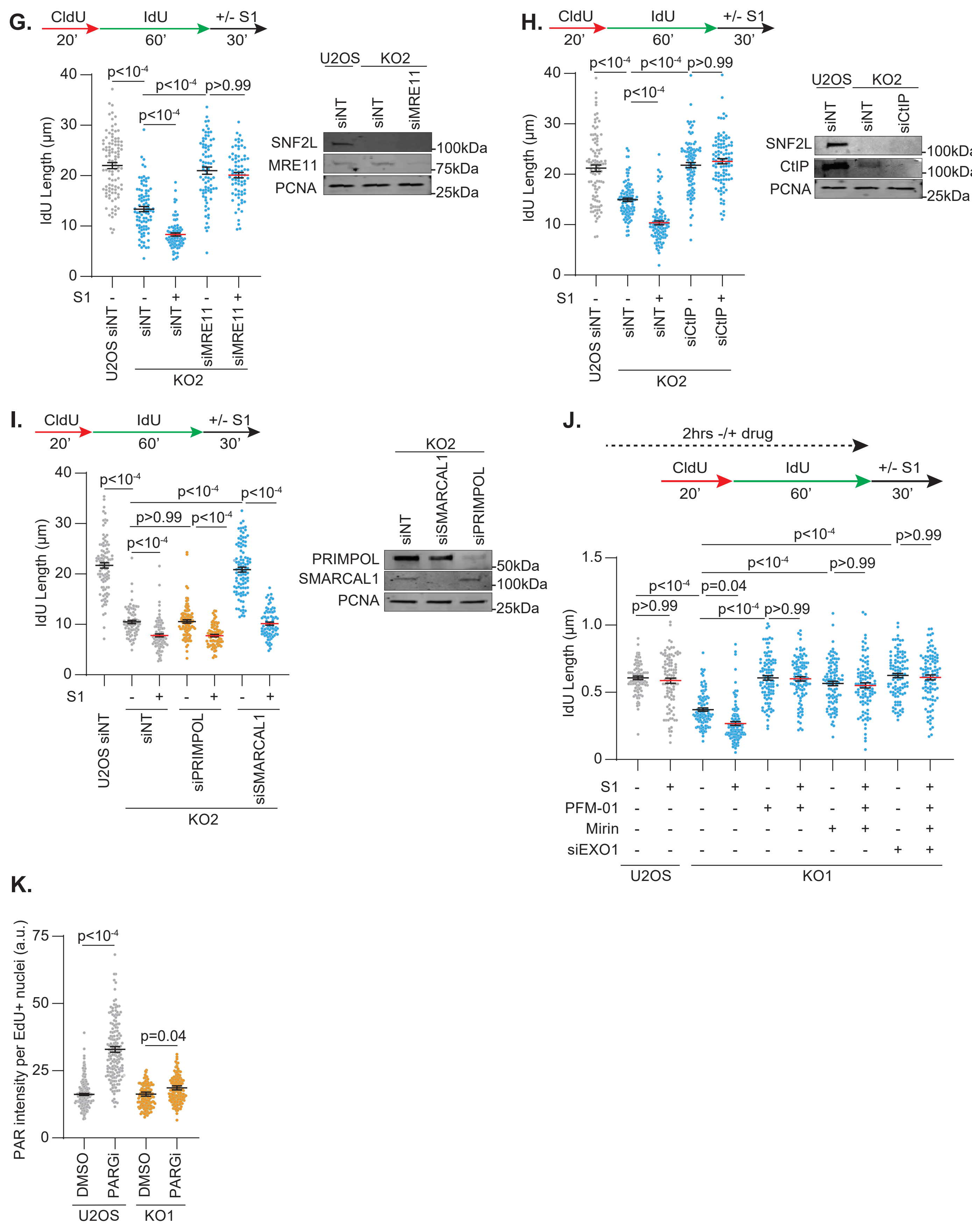

G.
CldU
IdU
+/- S1
20'
60'
30'
IdU Length (μm)
p<10-4
p>0.99
S1
U2OS siNT
siNT
siMRE11
KO2
U2OS
SNF2L
MRE11
PCNA
100kDa
75kDa
25kDa
H.
siCtIP
CtIP
I.
siPRIMPOL
siSMARCAL1
PRIMPOL
SMARCAL1
50kDa
J.
2hrs -/+ drug
p=0.04
PFM-01
Mirin
siEXO1
KO1
K.
PAR intensity per EdU+ nuclei (a.u.)
DMSO
PARGi

Figure S6

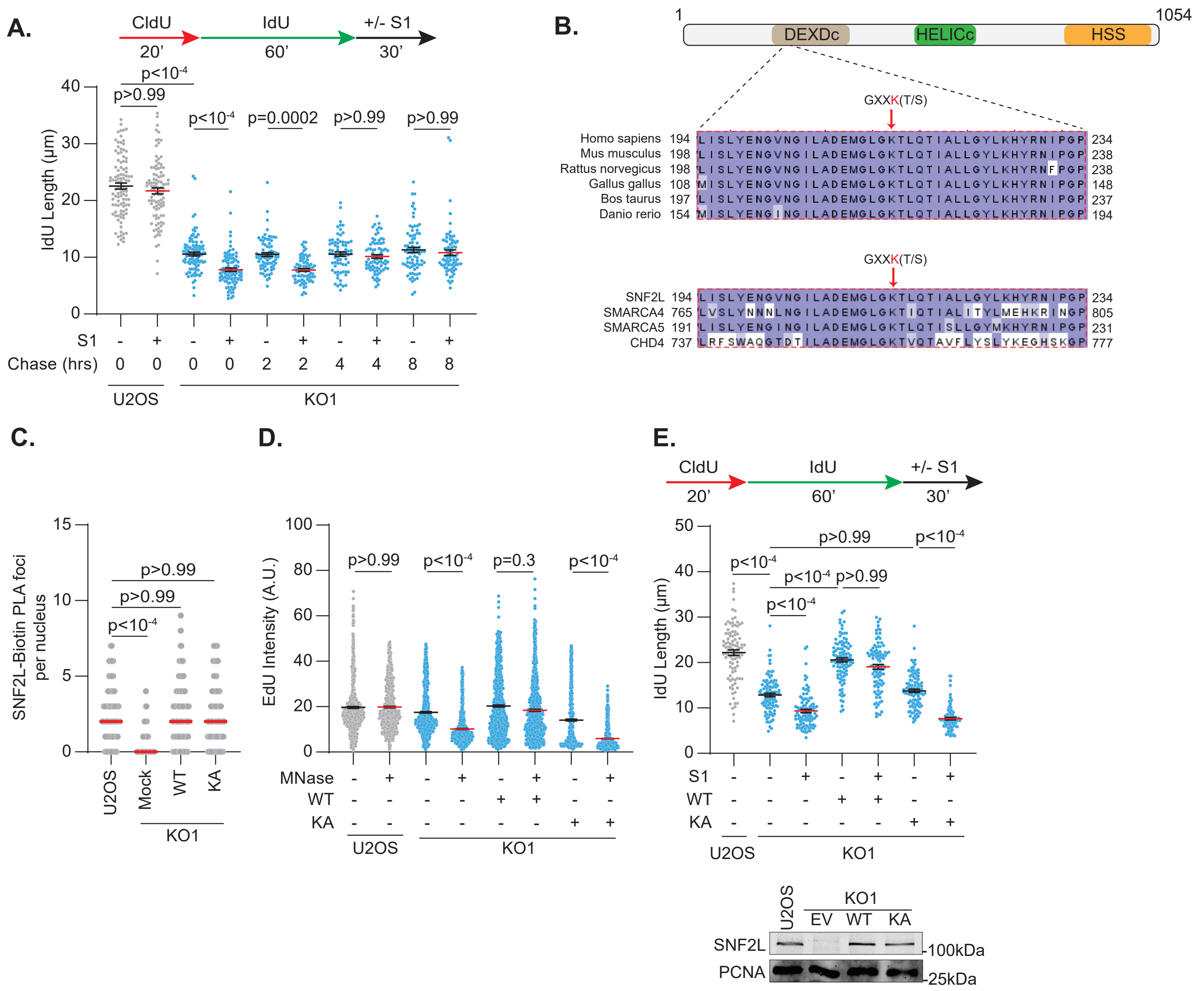
A.
CldU
20'
IdU
60'
+/- S1
30'
IdU Length (µm)
p<10-4
p>0.99
p<10-4
p=0.0002
p>0.99
p>0.99
S1
Chase (hrs)
U2OS
KO1
B.
1
1054
DEXDc
HELICc
HSS
GXXK(T/S)
Homo sapiens
Mus musculus
Rattus norvegicus
Gallus gallus
Bos taurus
Danio rerio
SNF2L
SMARCA4
SMARCA5
CHD4
C.
SNF2L-Biotin PLA foci per nucleus
p>0.99
p>0.99
p<10-4
U2OS
Mock
WT
KA
KO1
D.
EdU Intensity (A.U.)
p>0.99
p<10-4
p=0.3
p<10-4
MNase
WT
KA
U2OS
KO1
E.
CldU
20'
IdU
60'
+/- S1
30'
IdU Length (µm)
p>0.99
p<10-4
p<10-4
p<10-4
p>0.99
p<10-4
S1
WT
KA
U2OS
KO1
U2OS
KO1
EV
WT
KA
SNF2L
PCNA
100kDa
25kDa

SUPPLEMENTARY FIGURE AND TABLE LEGENDS

Figure S1 Replication elongation is dependent on SNF2L. (A) IdU/CldU ratios are plotted using dual labeled replication tracts from siNT- and siSNF2L-transfected U2OS cells. (B) siNT- and siSNF2L-transfected HeLa cells were subjected to DNA fiber analysis as shown. Immunoblot depicts SNF2L knockdown using KU70 as loading control. (C) Parental U2OS and multiple independent SNF2L knockout (KO) clones were subjected to DNA fiber analysis as shown. Immunoblot depicts SNF2L deletion using KU70 as loading control. (D) Immunoblot depicts SNF2L knockdown after 24 hours and 72 hours post siRNA transfection using KU70 as loading control. (E) Proliferation curves for U2OS cells transfected with siNT and siSNF2L are shown. (F) Cell viability of parental U2OS and three independent SNF2L KO clones were assessed using XTT proliferation assay. (G) Cell cycle distribution plots were obtained using flow cytometry for the indicated samples. Percentage distributions for U2OS and KO1 are included in the respective boxes. Black horizontal lines in panels A-C represent mean ± SEM. Data points in panels E and F represent mean ± SD. p-values were derived using Kruskal Wallis test with Dunn's multiple comparisons testing in panels A-C, and multiple unpaired t-tests in panels E and F. ns= not significant.

Figure S2 Loss of SNF2L induces fork collapse. (A) Tail moments were quantified using neutral comet assay in HEK293T and RPE-1 cells transfected with the indicated siRNAs. Cells treated with 1 µM camptothecin (CPT) for 1 hour serve as positive controls. (B) Tail moments were quantified using neutral comet assay in parental U2OS and SNF2L KO cells that were either mock transfected or transfected with SNF2L cDNA. (C) Percentage of pH3.S10 nuclei positive for EdU staining is depicted for the indicated samples. U2OS cells treated with 0.4 µM aphidicolin for 17 hours followed by release into complete media with 10 µM EdU for 30 minutes serves as a positive control. Bar graph represents mean ± SD. (D) IdU lengths of both arms at bidirectional replication tracts were measured using DNA fiber analyses. Individual lengths from bidirectional forks were plotted on both axes. Red lines have slope values of 0.5 and 2, illustrating boundaries of 50% deviation in bidirectional fork lengths, and percentage of forks outside of that range are shown on the graph. Red horizontal lines in box and whisker plots in panels A and B represent median. p-values were derived using Kruskal Wallis test with Dunn's multiple comparisons testing in panels A and B and multiple unpaired t-test in panel C.

Figure S3 Fork deceleration in SNF2L-deficient cells is dependent on replication fork reversal. (A) Immunoblot depicting knockdown of SMARCAL1, HLTF and ZRANB3 is shown from a single repeat using KU70 as loading control. (B) and (C) Parental U2OS or SNF2L KO cells transfected with the indicated siRNAs were subjected to DNA fiber analysis to measure IdU lengths using dual labeled replication tracts. (D) Immunoblot depicting knockdown of SMARCAL1 and HLTF is shown from a single repeat using KU70 as loading control. (E) Tail moments were quantified using neutral comet assay in U2OS and three KO clones transfected with either siNT or siSMARCAL1. Immunoblot depicts SMARCAL1 knockdown using PCNA as loading control. Black horizontal lines in panels B and C represent mean ± SEM. Red horizontal lines in box and whisker plots in panel E

represent median. p-values were derived using Kruskal Wallis test with Dunn's multiple comparisons testing in panels B, C and E.

Figure S4 SNF2L absence causes ssDNA gap accumulation. (A) Parental U2OS and SNF2L KO1 cells transfected with the indicated siRNAs were analyzed for exposed nascent IdU in native conditions. Mean IdU intensities from three biological replicates were normalized to siNT-transfected U2OS cells and plotted as bar graph. (B) Parental U2OS and SNF2L KO1 cells transfected with the indicated siRNAs were analyzed for exposed nascent IdU in native conditions. Immunoblot depicts knockdown of MUS81 using KU70 as loading control. (C) and (D) HeLa cells (panel C) and hTERT-RPE-1 cells (panel D) were transfected with the indicated siRNAs and subjected to DNA fiber analysis with or without S1 nuclease as per schematic. IdU lengths were measured using dual labeled replication tracts. (E) Parental U2OS or SNF2L KO1 cells were transfected with the indicated siRNAs and subjected to DNA fiber analysis with or without S1 nuclease as per schematic. IdU lengths were measured using dual labeled replication tracts. Immunoblot depicts knockdown of SNF2H using KU70 as loading control. Bar graph in panel A represents mean $\pm$ SD. Horizontal lines in panels B-E represent mean $\pm$ SEM. p-values were derived using unpaired t-test in panel A and Kruskal Wallis test with Dunn's multiple comparisons testing in panels B-E**.**

Figure S5 Nascent DNA gap accumulation when SNF2L is removed is dependent on nucleases MRE11 and EXO1, but independent of PRIMPOL. (A) Parental U2OS and SNF2L KO1 cells transfected with the indicated siRNAs were analyzed for exposed parental IdU in native conditions. (B) FBH1 knockout (KO) cells were transfected with the indicated siRNAs and subjected to DNA fiber analysis with or without S1 nuclease as per schematic. (C) – (E) Parental U2OS or SNF2L KO1 cells were subjected to DNA fiber analysis with or without S1 nuclease as per schematic. Cells were either untransfected or transfected with the indicated siRNAs. (F) Parental U2OS and SNF2L KO1 cells treated with either DMSO or mirin and analyzed for exposed nascent IdU in native conditions. (G) – (I) Parental U2OS or SNF2L KO2 cells were transfected with the indicated siRNAs and subjected to DNA fiber analysis with or without S1 nuclease as per schematic. Respective immunoblots depict knockdown efficiencies using PCNA as loading control. (J) Parental U2OS or SNF2L KO1 cells were transfected with the indicated siRNAs and subjected to DNA combing procedure with or without S1 nuclease as per schematic. Where indicated, cells were pre-treated with either mirin or PFM-01 for 2 hours. IdU lengths were measured using dual labeled replication tracts. (K) Parental U2OS or SNF2L KO1 cells were treated with either DMSO or 10μM PARGi for 30 minutes prior to immunostaining with PAR antibody. Horizontal lines represent mean $\pm$ SEM. p-values were derived using Kruskal Wallis test with Dunn's multiple comparisons testing in all panels**.**

Figure S6 Aberrant nucleosome assembly on nascent DNA triggers gap formation when SNF2L is absent. (A) Parental U2OS or SNF2L KO cells were subjected to DNA fiber analysis with S1 nuclease as per schematic to detect gaps in nascent and mature DNA. Where indicated, cells were released into IdU-free media for 2, 4 or 8 hours to test sensitivity of mature chromatin to S1 nuclease. IdU lengths were measured using dual

labeled replication tracts. (B) Protein domain map for SNF2L is shown (DEXDc= DEAD-like helicases superfamily, HELICc= Helicase superfamily c-terminal domain, HSS= HAND-SANT-SLIDE domain). In the top panel, sequence alignment of SNF2L from the indicated organisms is shown using JalView. Catalytic isoform of SNF2L lacking internal exon 13 was selected for *Homo sapiens*. Shades of blue represent BLOSUM62 scores to indicate degree of sequence identity. In the bottom panel, sequence alignment of SNF2L to other nucleosome remodelers in humans is depicted. The conserved ATP-binding motif GXXK(T/S) (1) is highlighted wherein mutation of conserved lysine abrogates ATPase activity in SMARCA4 (2), SMARCA5 (3) and CHD4 (4). Both panels show GXXK(T/S) motif in which lysine residue (red) was mutated to alanine to generate SNFL-K214A mutant. (C) SNF2L proximity to nascent DNA was detected using SIRF assay in either parental U2OS cells or SNF2L KO cells transfected with the indicated vectors. Antibodies targeting SNF2L and biotinylated EdU were utilized to detect proximity of SNF2L to nascent DNA. (D) Parental U2OS and SNF2L KO cells were pulsed with EdU for 60 minutes, permeabilized and were either untreated or treated with MNase for 30 minutes prior to fixation. EdU intensities were measured by quantitative imaging. A.U.= Arbitrary Units. (E) Stable SNF2L KO1 cell lines generated using empty vector, SNF2L-WT or SNF2L-K214A cDNA were subjected to DNA fiber analysis with or without S1 nuclease as per schematic. Parental U2OS cells were included for comparison. Immunoblot validating SNF2L expression is depicted using PCNA as loading control. Horizontal lines in panel A, D and E represent mean ± SEM. Red horizontal lines in panel C represent median. p-values were derived using Kruskal Wallis test with Dunn’s multiple comparisons testing in all panels.

Table S1 List of siRNA oligos used in this study.

siRNAs	Sequence	Source
Control siRNA	AllStars negative control siRNA	Qiagen, Cat# SI03650318
SMARCA1	5'-GGCGAUACCUGGUCAUUGA-3'	Horizon, Cat# J-011392-08
BPTF	5'-GAACCGAAGACUCAUAAUA-3';<br>5'-GGUCCAACUUGCAGAAUUA-3';<br>5'-GCUCUUAGAUUCUGACAGU-3';<br>5'-GAAUUAAGGGUAUAGGAAA-3'	Horizon, Cat# L-004025-00
MUS81	5'-GGGUAUACCUGGUGGAAGA-3'	Horizon, Cat# D-016143-04
MRE11	5'-GAAAGGCUCUAUCGAAUGU-3'	Horizon, Cat# J-009271-08
SMARCAL1	5'-GCUUUGACCUUCUUAGCAA-3'	Horizon, Cat# J-013058-06
ZRANB3	5'-GAUCAGACAUCACACGAUU-3'	Horizon, Cat# D-010025-03
HLTF	5'-CCAGAUGACUUUCUAACUA-3';<br>5'-GAUAGAGAAUGGUGGCAUA-3';<br>5'-GCAGGAUCUUCUAAGGUUA-3';<br>5'-GGAUUUGUGUUUACUCGUU-3'	Horizon, Cat# L-006448-00
RECQL1	5'-GAGCUUAUGUUACCAGUUA-3';<br>5'-CUACGGCUUUGGAGAUAUA-3';<br>5'-GAUUAUAAGGCACUUGGUA-3';<br>5'-GGGCAAGCAAUGAAUAUGA-3'	Horizon, Cat# L-013597-00
SMARCA5	5'-GGAAUGGUAUACUCGGAUA-3';<br>5'-GGGCAAAUAGAUUCGAGUA-3';<br>5'-GGAUUUACCAAUUGGAAUA-3';<br>5'-GUUCUUUCCUCCACGUUUA-3'	Horizon, Cat# L-011478-00
PRIMPOL	5'-GAGGAAAGCUGGACAUCGA-3';<br>5'-GGAGAUGGACAACGUAUUU-3';<br>5'-GCAAAUUGAAGAACGAGCA-3';<br>5'-AAGAUGUUUCUGACGAAUA-3'	Horizon, Cat# L-016804-02
RBBP8/CtIP	5'-GGAGCUACCUCUAGUAUCA-3';<br>5'-GAGGUUAUAUUAAGGAAGA-3';<br>5'-GAACAGAAUAGGACUGAGU-3';<br>5'-GCACGUUGCCCAAAGAUUC-3'	Horizon, Cat# L-011376-00
EXO1	5'-GCACGUAAUUCAAGUGAUG-3';<br>5'-GUAAAUGGACCUACUAACA-3';<br>5'-CCACCUAGGACGAGAAAUA-3';<br>5'-CGGAAGAGAAGUUUCGUUA-3'	Horizon, Cat# L-013120-00

SUPPLEMENTARY REFERENCES

1. Tanner, N.K. and Linder, P. (2001) DExD/H box RNA helicases: from generic motors to specific dissociation functions. *Mol Cell*, **8**, 251-262.
2. Stanton, B.Z., Hodges, C., Calarco, J.P., Braun, S.M., Ku, W.L., Kadoch, C., Zhao, K. and Crabtree, G.R. (2017) Smarca4 ATPase mutations disrupt direct eviction of PRC1 from chromatin. *Nat Genet*, **49**, 282-288.
3. Brestovitsky, A., Sharf, R., Mittelman, K. and Kleinberger, T. (2011) The adenovirus E4orf4 protein targets PP2A to the ACF chromatin-remodeling factor and induces cell death through regulation of SNF2h-containing complexes. *Nucleic Acids Res*, **39**, 6414-6427.
4. Pan, M.R., Hsieh, H.J., Dai, H., Hung, W.C., Li, K., Peng, G. and Lin, S.Y. (2012) Chromodomain helicase DNA-binding protein 4 (CHD4) regulates homologous recombination DNA repair, and its deficiency sensitizes cells to poly(ADP-ribose) polymerase (PARP) inhibitor treatment. *J Biol Chem*, **287**, 6764-6772.
